# Lead Halide Perovskite Nanocrystals in Metal‐Organic Frameworks: Synthesis, Properties, and Applications

**DOI:** 10.1002/advs.202505407

**Published:** 2025-08-30

**Authors:** Xiaochen Fang, Zhijie Xie, Swellam Sharshir, Hanhui Lei, Jiaxian Zheng, Biao Zheng, Xiangfeng Lin, Xiaoteng Liu, Zhanhui Yuan

**Affiliations:** ^1^ College of Materials Engineering Fujian Agriculture and Forestry University Fuzhou 350002 China; ^2^ Mechanical Engineering Department Faculty of Engineering Kafrelsheikh University Kafrelsheikh 33516 Egypt; ^3^ Department of Mechanical and Construction Engineering Northumbria University Newcastle upon Tyne NE1 8ST UK; ^4^ Fujian Key Laboratory of Functional Marine Sensing Materials College of Material and Chemical Engineering Minjiang University Fuzhou 350108 China

**Keywords:** applications, halide perovskite nanocrystals, metal‐organic framework, properties

## Abstract

Halide perovskite nanocrystals (PeNCs) with their exceptional light absorption and tunable bandgap, are promising candidates for solar energy harvesting and high‐performance photodetectors. However, the susceptibility of PeNCs to degradation limits their widespread application. The integration of halide perovskites within metal‐organic frameworks (MOFs) has recently garnered significant attention as a strategy to create composite materials. The above encapsulation enhances the stability of perovskites against environmental stressors, such as humidity, temperature, and light, leading to improved performance characteristics. This paper provides a comprehensive review of perovskite–metal–organic framework composites (PeMOFs) and their applications in photoelectric conversion. It explores the various preparation methods for PeMOFs and highlights recent advancements in the field. The review examines the specific functions of MOFs within these composite materials and summarizes recent progress in a range of applications, including photocatalysis, sensing, light‐emitting diodes, perovskite solar cells, luminescent anti‐counterfeiting, information security, and detection. Finally, the paper discusses potential future trends and emerging applications for PeMOFs.

## Graphical abstract

Perovskites, when applied in devices, face challenges like stability. Integrating halide perovskites into metal‐organic frameworks (MOFs) as composites has gained attention. This approach can enhance stability, facilitate electron transport, and improve crystallinity, thus boosting perovskite performance in various applications.

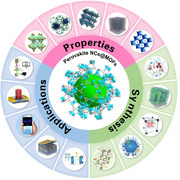

## 1 Introduction

**Figure 1:**
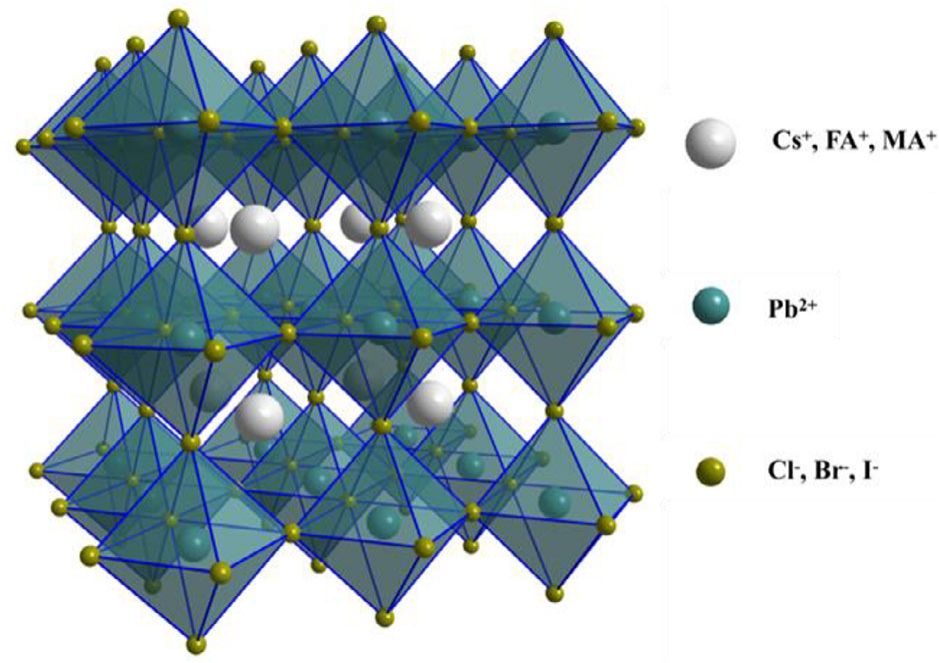
Schematic depiction of cubic phase PeNCs.

Perovskite nanocrystals (PeNCs) combined with lead halides have recently garnered significant attention as promising materials for photovoltaics and optoelectronics.^[^ ^1^, ^2^, ^3^, ^4^, ^5^ ^]^ These materials can be classified as metal halide perovskites, with the basic structure ABX_3_, where A represents a monovalent cation, B represents a metal cation, and X represents a halide anion (**Figure** **1**).^[^ ^6^ ^]^ Despite the lack of shell and surface engineering, the newly developed colloidal PeNCs appear to be better than established cadmium‐based PeNCs for their narrow full width at half maximum (FWHM) and relatively high photoluminescence quantum yield (PLQY) (even up to 100%).^[^ ^7^ ^]^ These are mainly owed to their closely packed crystal lattice, point defects, and good surface passivation to suppress non‐radiative recombination losses. Owing to these unique properties, PeNCs demonstrate superior efficiency and stability compared to cadmium‐based NCs, making them particularly suitable for various optical applications including display technologies, lighting systems, and other promising fields. Furthermore, different parts and sizes of PeNCs may successfully change their bandwidth and photoluminescence.^[^ ^8^, ^9^, ^10^ ^]^

The vulnerability of these materials to degradation caused by heat, light, and humidity restricts their commercial application. Consequently, encapsulating PeNCs within a protective host matrix has emerged as a viable strategy to mitigate this limitation.^[^ ^11^, ^12^ ^]^ To enhance the stability of PeNCs, coating them with materials such as metal‐organic frameworks (MOFs), polymers, or inorganic oxides is highly recommended. This coating serves as a protective barrier against degradation induced by heat, oxygen, and moisture. This protective layer effectively prevents PeNCs degradation and mitigates chemical reactions that could compromise their optical properties. Furthermore, the coating is effective in preventing the desorption of surface ligands, thereby ensuring the stability of the nanocrystals and preventing agglomeration. By preserving the ligands and providing environmental protection, the coating significantly enhances the stability and extends the lifetime of PeNCs in various applications.

**Figure 2:**
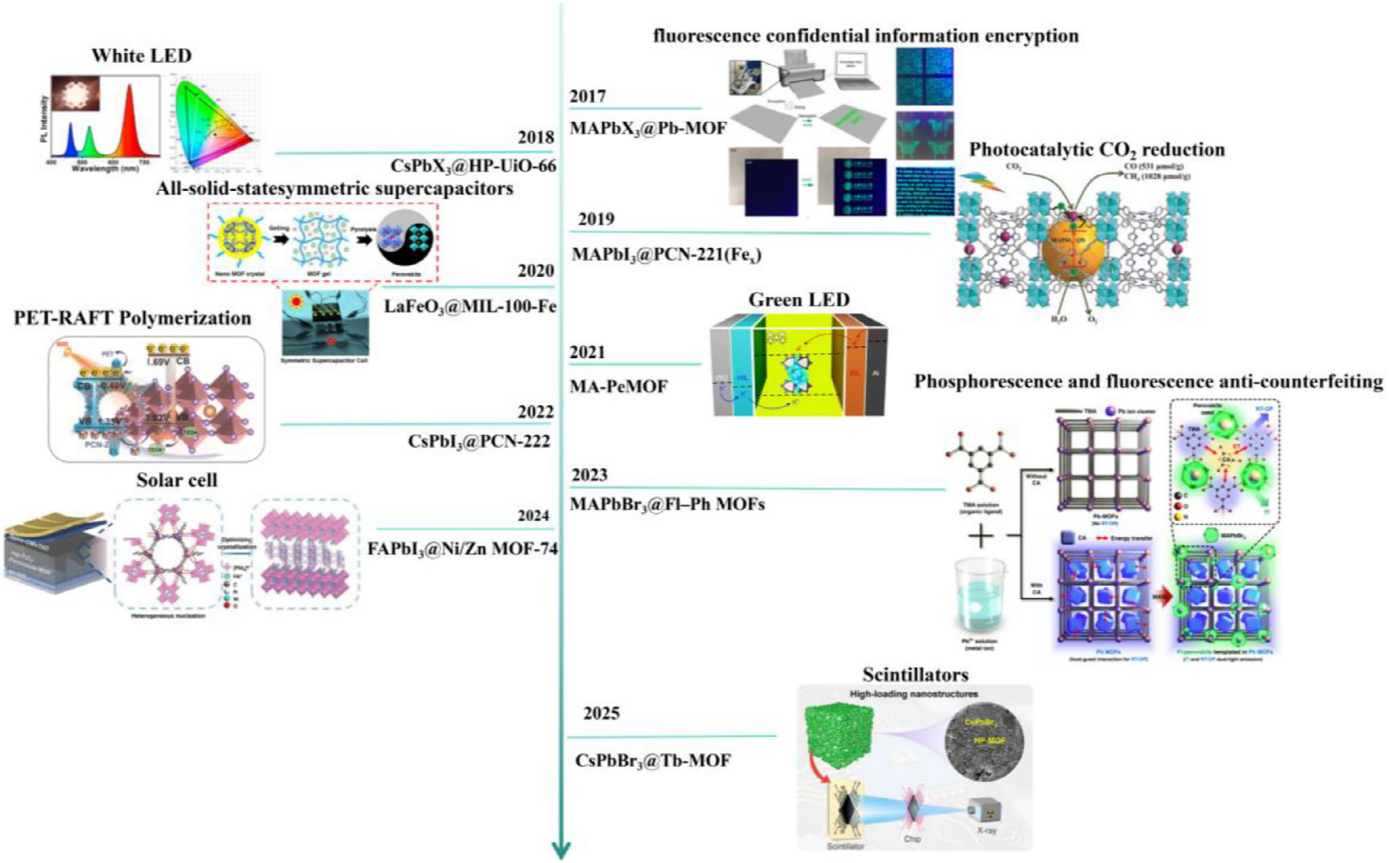
Timeline of PeMOFs composite materials. Reproduced with permission.^[^ ^20^, ^21^, ^22^, ^23^, ^24^, ^25^, ^26^, ^27^, ^28^ ^]^ Copyright 2017, Springer Nature. Copyright 2018, Elsevier. Copyright 2019 willey. Copyright 2020 Elsevier. Copyright 2021 Springer. Copyright 2022 Wiley. Copyright 2023 Springer Nature. Copyright willey 2024. Copyright American Chemical Society 2025.

MOFs are innovative porous crystalline materials assembled from metal‐containing nodes and multifunctional organic linkers via coordination,^[^ ^13^ ^]^ exhibiting high surface areas, tunable pore sizes, and tailorable functionality.^[^ ^14^ ^]^ Therefore, MOFs are widely used in applications including lighting, gas adsorption and storage, catalysis, sensing, molecular separation, as well as protective encapsulation matrices for PeNCs.^[^ ^15^, ^16^, ^17^, ^18^, ^19^ ^]^ MOFs are also increasingly utilized as protective encapsulation matrices for PeNCs. These novel perovskite‐MOF composites (PeMOFs) offer enhanced stability and introduce new functionalities, enabling applications such as photocatalysis, sensing, detection, and information security (**Figure** **2**).^[^ ^20^, ^21^, ^22^, ^23^, ^24^, ^25^, ^26^, ^27^, ^28^ ^]^ Based on the previous research context^[^ ^29^, ^30^, ^31^, ^32^, ^33^, ^34^ ^]^ this review more specifically summarizes the recent advances in the structure of PeMOFs and their applications in various photonic devices. Specifically, we will discuss the role of MOFs in addressing challenges associated with perovskites in various applications, and explain how PeMOFs offer potential solutions.

## 2 Strategies for Improving the Optical Property of PeNCs

Perovskite nanocrystals (PeNCs) possess several attractive properties, including a high light absorption coefficient, a tunable bandgap, long carrier diffusion lengths, and low carrier recombination rates. However, their inherent instability, largely due to their ionic crystal structure, poses a significant obstacle to their broader application. PeNCs are particularly susceptible to degradation when exposed to water, humidity, light, and elevated temperatures. The following section will review recent advances in strategies designed to improve both the performance and stability of PeNCs.

### 2.1 Ion Doping Engineering

Doping is an effective strategy for tuning the optoelectronic properties of semiconductor materials. Incorporating trace amounts of impurity atoms into semiconductors significantly affects their stability and optoelectronic performance, as demonstrated by research.^[^ ^35^, ^36^ ^]^ A variety of dopants are currently employed in PeNCs, primarily including transition metals, main group metals, and rare earth metal cations.^[^ ^37^, ^38^ ^]^ The subsequent section will review recent advances in understanding the effects of ion doping on the performance and stability of PeNCs.

#### 2.1.1 A‐Site Cation Engineering

A‐site cation engineering in perovskite nanocrystals involves the substitution or doping of cations at the A‐site, which are typically organic or inorganic within the perovskite structure. In lead halide perovskites with the formula APbX_3_ (where A = Cs, MA, FA and X = Cl, Br, I), Cs^+^ is the only inorganic cation capable of occupying the A‐site. This is attributed to its relatively large ionic radius, which contributes to the structural stability of the crystalline perovskite lattice.^[^ ^39^, ^40^ ^]^ However, doping PeNCs with other monovalent cations can effectively modulate their structure, leading to improved optoelectronic properties and enhanced stability. Specifically, when dopant ions occupy the A‐site, they alter the average ionic radius, increase octahedral distortion, and reduce lattice symmetry. These changes can lower the substitution energy and ultimately enhance luminescence performance.^[^ ^41^, ^42^ ^]^ To enhance the photostability and water stability of perovskite solar cells, Prasanna et al. partially substituted CH(CH_2_)_2_^+^ (FA^+^) with Cs^+^. This substitution reduced the size of the perovskite octahedra and significantly strengthened the interionic interactions. The improved ionic coupling enhanced the perovskite material's overall stability, making it less susceptible to degradation from light and moisture.^[^ ^43^ ^]^ Zhao et al. achieved highly stable perovskite light‐emitting diodes (PeLEDs) through dual doping of FAPbI_3_ with Cs^+^ and Rb⁺.^[^ ^44^ ^]^ Their findings revealed that Cs⁺ ions were uniformly distributed within the perovskite matrix, whereas Rb⁺ ions preferentially segregated to the surface and grain boundaries. The incorporation of Cs⁺ and Rb⁺ increased the net atomic charge of neighboring anions and strengthened the Coulombic interactions between cations and the inorganic framework. This dual doping strategy effectively suppressed the formation of iodide vacancies and impeded I^−^ migration both within the perovskite matrix and along grain boundaries. Beyond Cs⁺ and Rb⁺, other A‐site dopants, such as MA^⁺^, Na^⁺^, and K^⁺^, are also commonly employed in perovskites to significantly enhance material stability and optical properties.^[^ ^45^, ^46^, ^47^ ^]^

#### 2.1.2 B‐Site Cation Engineering

**Figure 3:**
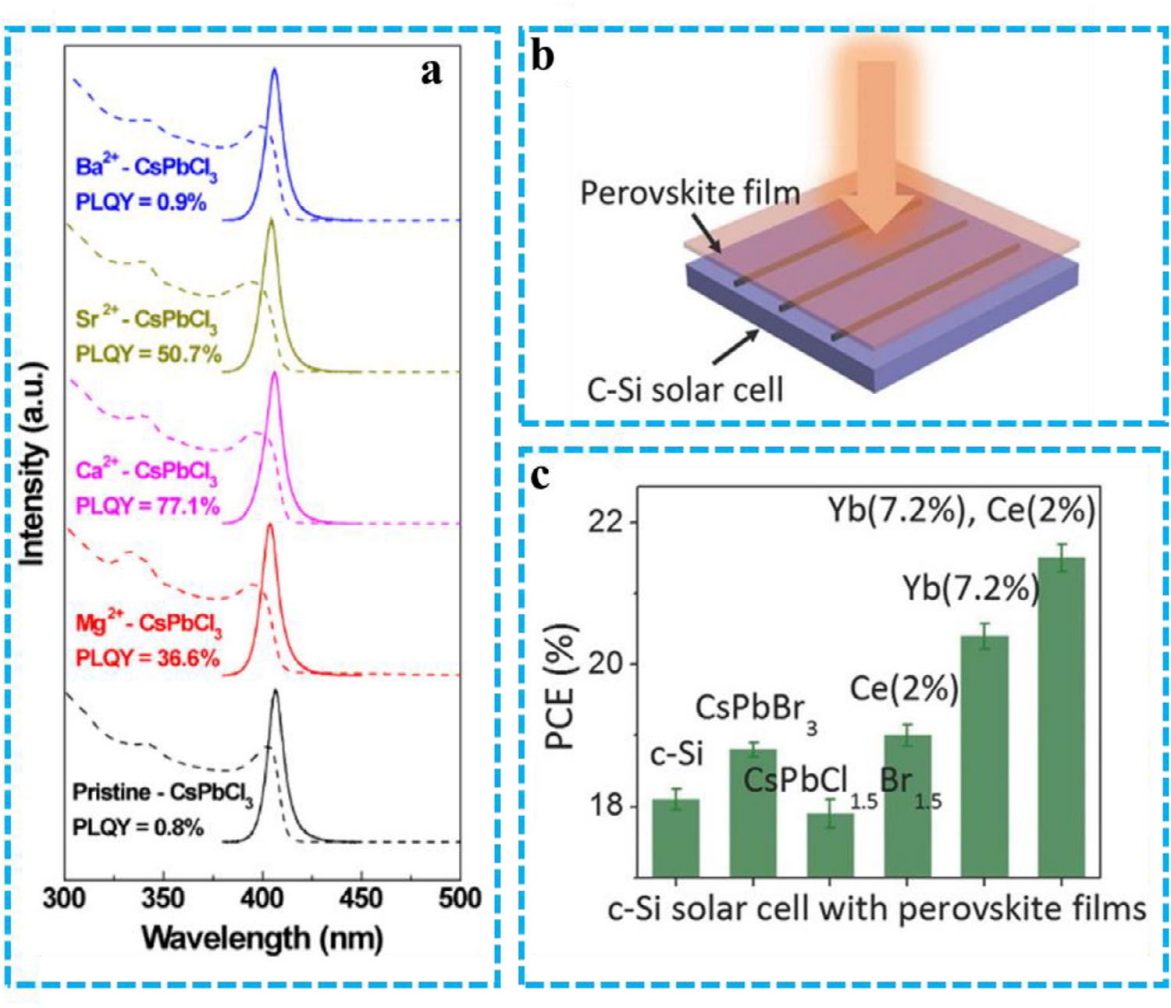
a) The PL (solid line) and UV‐vis absorption (dotted line) spectra of pristine and alkaline‐earth‐CsPbCl_3_. Reproduced with permission.^[^ ^52^ ^]^ Copyright 2019 American Chemical Society. b) Schematic diagram of perovskite film, c) PCE of SSCs coated with different PeNCs. Reproduced with permission.^[^ ^54^ ^]^ Copyright 2017 Wiley.

B‐site doping in PeNCs, similarly, can significantly enhance the properties of perovskites. Given lead's crucial structural role in metal halide perovskites and its substantial influence on electronic states, doping at the B‐site is particularly important.^[^ ^48^ ^]^ The most widely studied dopants include Mn^2+^, Co^2+^, and other transition metals.^[^ ^49^, ^50^ ^]^ In addition, researchers have explored the use of other metal ions, such as Mg^2+^ and Cu^2+^, as dopants. For example, Das et al. successfully doped Mg^2+^ into CsPbCl_3_ and CsPbBr_3_ PeNCs by adding MgCl_2_ to the PeNCs dispersed in toluene solution. This doping process significantly enhanced the photoluminescence quantum yield (PLQY) from 51 to 100%. Furthermore, the increased occupancy of Mg^2+^ improved the stability of the PeNCs and their resistance to polar solvents, making them more suitable for optoelectronic applications.^[^ ^51^ ^]^ Similarly, Chen et al. incorporated Ca^2+^ and Sr^2+^ ions into PeNCs and found that these ions were primarily located on the surface of the PeNCs, forming a passivation layer that reduced radiative recombination, thereby minimizing losses of photogenerated charge carriers.^[^ ^52^ ^]^ Under optimal CaCl_2_ addition during synthesis, the PLQY of violet‐emitting CsPbCl_3_ NCs reached ≈77.1%, which was significantly higher than that of PeNCs synthesized without any foreign cations. The stability of the PeNCs was also further improved (**Figure** **3**a).

Rare earth (RE) ions are commonly used as dopants. Doping II–VI group semiconductor materials and oxides with rare earth elements enhances properties like photoluminescence, charge transport, and energy transfer.^[^ ^53^ ^]^ Incorporating RE ions into PeNCs can introduce additional energy levels within the bandgap, enabling finer control over emitted light. This makes RE‐doped PeNCs promising candidates for applications in lighting, displays, lasers, and other optoelectronic devices. Doping with rare‐earth ions is a well‐established strategy for enhancing the optoelectronic and optical properties of materials. The closely spaced, discrete energy levels characteristic of RE ions allow them to produce narrowband emissions spanning the UV to infrared (IR) regions of the spectrum. This characteristic enables RE ions to act as key determinants of the luminescence performance of lead halide perovskites, thereby broadening their applicability in optoelectronic devices. Due to their smaller ionic radii compared to lead ions, doping PeNCs with RE ions induces lattice contraction. This contraction enhances the stability of the PeNCs and consequently improves the PLQY. Furthermore, RE ions are characterized by a rich array of energy levels and stable optical properties. For instance, Zhou et al. demonstrated that doping CsPbX_3_ PeNCs with Ce^3+^ and Yb^3+^ enhanced the PLQY of excitons. The incorporation of these rare earth ions introduces additional energy transfer pathways within the system, promoting radiative recombination of excitons.^[^ ^54^ ^]^ The quantum‐cutting effect in rare earth elements contributes to their high PLQY, leading to significant improvements in their optical properties. Consequently, the doped PeNCs were effectively used as down‐converters for commercial silicon solar cells (SSCs) (Figure 3b), resulting in an enhancement of the SSCs' power conversion efficiency (PCE) from 18.1 to 21.5% (Figure 3c). Although rare‐earth doping offers some improvements to the optical, optoelectronic, and stability properties of perovskites, it is insufficient to enable their operation under harsh conditions such as high temperature and humidity. Therefore, ligand modification and surface passivation strategies have recently garnered considerable research interest.

### 2.2 Ligand Engineering

**Figure 4:**
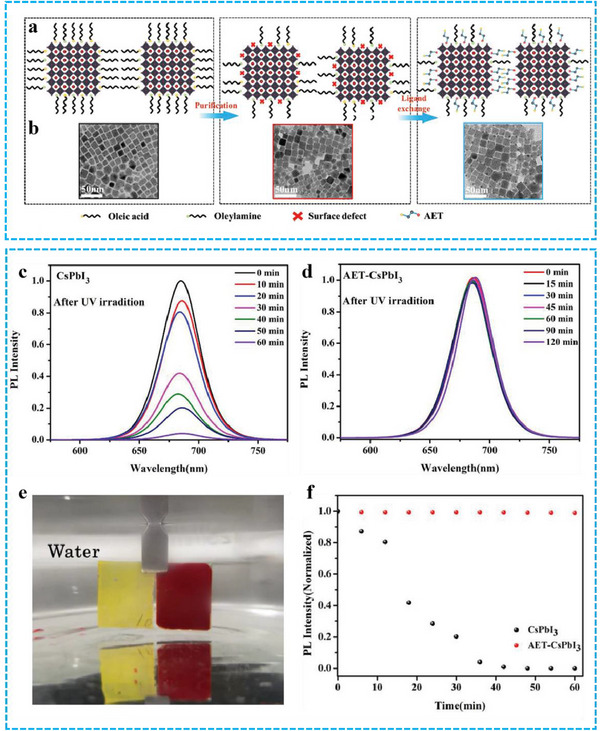
a) Schematic diagram of purification and AET ligand exchange b) TEM images of CsPbI_3_ in its initial state, after Methoxyacetic acid (Me) Acetic Acid (OAc) purification, and after AET treatment, c,d) PL spectra under UV light irradiation at different times, e,f) Images and PL spectra after soaking in water for different durations. Reproduced with permission.^[^ ^56^ ^]^ Copyright 2019 Wiley.

Ligands play a crucial role in enhancing both the thermal stability and optoelectronic performance of perovskite materials. Ligand engineering offers a versatile approach to optimize perovskite crystallization, minimize surface defects, improve overall stability, and tailor optoelectronic properties. This strategy provides effective pathways for developing high‐performance and stable PeNCs optoelectronic devices.^[^ ^55^ ^]^ Bi et al. demonstrated that coating CsPbI_3_ films with 2‐aminoethanethiol (AET‐CsPbI_3_) yields stable PeNCs with high PLQY while preserving their morphology (**Figure** **4**a,b).^[^ ^56^ ^]^ The AET ligand effectively passivates surface traps on the PeNCs, leading to enhanced PL efficiency. Significantly, AET‐CsPbI_3_ retained over 95% of its initial photoluminescence intensity in both solution and film phases, even after 2 h of UV irradiation and 1 h of water exposure (Figure 4c–f). Although ligand engineering can enhance PeNCs' stability, it cannot change their ionic crystal nature, which still causes poor stability. Therefore, surface coating offers a further way to boost their high temperature and humidity stability.

### 2.3 Surface Coating Engineering

Encapsulating PeNCs within materials such as polymers, inorganic oxides, or metal‐organic frameworks offers a 2‐fold benefit: it shields the PeNCs from detrimental environmental factors like moisture, oxygen, and light, and it minimizes the loss of surface ligands. This protective encapsulation ensures the structural integrity of the PeNCs, preventing degradation and promoting the long‐term stability required for sustained performance.

#### 2.3.1 Silica Encapsulation

Silica nanoparticles, as one of the most common inorganic nanomaterials, are not only widely available but also possess excellent characteristics such as chemical stability, low cost, high‐temperature resistance, colorless transparency, and environmental friendliness.^[^ ^57^ ^]^ Therefore, Silica is widely employed to encapsulate luminescent materials. Notably, Liu et al. fabricated CsPbX_3_@mesoporous silica (mSiO_2_) composites using a straightforward stirring method, resulting in materials with high thermal stability. Specifically, these composites maintained 40% of their original luminescence intensity at 100 °C.^[^ ^58^ ^]^ Shi et al. synthesized CsPbX_3_@mSiO_2_ composites via a sol‐gel method, demonstrating that they maintained over 50% of their initial luminescence after exposure to water and ethanol for 60 min.^[^ ^59^ ^]^ The PeNCs encapsulated in silica exhibits good stability. However, PeNCs aggregation within mSiO_2_ can occur, and the mSiO_2_ matrix can influence its conductivity, consequently affecting the perovskite's luminescence and optoelectronic properties.

#### 2.3.2 Upconversion Nanoparticles Encapsulation

Researchers have subsequently proposed coating perovskites with upconversion nanoparticles (UCNPs). The heterojunction between UCNPs and PeNCs improves the optoelectronic performance of perovskite materials. The inherent bandgaps of perovskite nanocrystals (PeNCs), typically exceeding 1.5 eV, limit their responsiveness to near‐infrared (NIR) light, thus restricting their application in various advanced technologies. To address this limitation, integrating PeNCs with lanthanide‐doped UCNPs presents a promising strategy. UCNPs efficiently convert low‐energy NIR photons into higher‐energy UV and visible photons. This energy transfer from UCNPs to PeNCs not only extends the NIR response range of the composite material but also introduces a novel emission incorporating UCNPs profile luminescence. This profile offers multidimensional tunability (e.g., wavelength, lifetime, and polarization) under low‐ to medium‐power NIR excitation. Consequently, this synergistic combination overcomes the individual limitations of both PeNCs and UCNPs, opening up new avenues for materials design and device engineering.^[^ ^60^ ^]^ Ruan et al. used NaYF_4_:Yb,Tm as an intermediate phase to incorporate CsPbBr_3_, forming CsPbBr_3_–NaYF_4_:Yb,Tm composites with a watermelon‐like heterostructure.^[^ ^61^ ^]^ These hybrid composites demonstrate not only NIR excitability but also enhanced stability. Specifically, the CsPbBr_3_–NaYF_4_:Yb,Tm NCs exhibited significantly improved thermal stability, retaining 71% of their initial fluorescence intensity after exposure to 200 °C. Under 980 nm excitation, the relative fluorescence intensity of these NCs remained at ≈90% throughout the heating process. Furthermore, in a cyclohexane and ethanol (9:1) mixed solvent, the CsPbBr_3_–NaYF_4_:Yb,Tm NCs retained 93% of their fluorescence intensity after 10 min and 85% after 120 min under identical conditions. These results indicate that UCNPs can substantially enhance the stability of perovskites while simultaneously providing additional UCNP luminescence. MOFs have also been observed to exhibit similar stabilizing properties.

#### 2.3.3 MOFs Encapsulation

MOFs are distinguished by their high porosity, large surface area, and diverse structures. Made of organic ligands and inorganic metal ions, these porous materials excel in applications like biomedicine, electronics, gas storage, catalysis, sensing, and lighting. MOFs are ideal for enhancing the stability of PeNCs, mitigating photoluminescence quenching and aggregation. Their large surface area and active sites boost PeNCs' catalytic activity. Zhang et al. used direct conversion to synthesize PeNCs within MOF pores, an innovative method impossible with traditional materials. This novel composite, with modifiable organic linkers and metal nodes, enables new applications such as data encryption, metal ion detection in water, and multiphoton‐excited upconversion luminescence.

## 3 Growth of PeNCs within MOFs (PeMOFs)

This chapter provides a comprehensive and systematic review of PeMOFs preparation methods, analyzing their respective advantages and disadvantages. It further explores the influence of preparation processes on PeMOFs structure and properties and outlines future trends in PeMOFs synthesis. The aim is to offer novel insights and methodologies for PeMOFs preparation and application, thereby fostering advancements in the development and practical utilization of this material.

### 3.1 Brief Introduction of MOFs

MOFs, crystalline porous materials discovered in 1995, have rapidly become a versatile class of materials. Their construction relies on organic ligands, including carboxylate, polynitrogen, and other negatively charged types, enabling applications across diverse fields.^[^ ^62^ ^]^ MOFs are advantageous due to their high porosity, large specific surface area, and tunable physicochemical properties. These distinctive attributes enable MOFs to be applied across a wide spectrum of fields, including catalysis, sensing, gas storage and separation, biomedical applications, energy storage and conversion, and environmental remediation. MOFs function as adaptable host matrices for incorporating diverse guest compounds. The inclusion of these guests within the MOF structure results in functional composites exhibiting novel properties and improved performance. A broad array of materials has been investigated as potential guest species, encompassing metal oxides, silica, quantum dots, organic polymers, polyoxometalates, biomolecules, carbon materials, graphene, and even other MOFs. The resulting synergistic host‐guest interactions give rise to emergent properties that exceed the capabilities of the individual constituents.

### 3.2 Synthesis of PeMOFs

Integrating PeNCs into MOFs offers a promising approach to enhance the optical and optoelectronic properties of PeNCs–MOF composites. Based on the relative timing of PeNCs and MOF formation, PeMOFs synthesis strategies can be broadly categorized into “bottle‐around‐ship” and “ship‐in‐bottle” approaches.

#### 3.2.1 Ship‐in‐Bottle

The “ship‐in‐bottle” method involves the nucleation and growth of PeNCs within the pre‐existing pores of MOFs, resulting in the formation of PeMOFs. Crucially, the MOF framework dictates the spatial positioning and size of the perovskite nanocrystals, leveraging the framework's defined structure as a template for nanocrystal growth. The inherent porosity of MOFs facilitates the confined growth of PeNCs, promoting uniform size distribution and preventing aggregation. This confinement is vital, as aggregation often leads to diminished optical properties and performance. By controlling the size and dispersion of PeNCs, MOFs enhance their stability, uniformity, and overall functionality, making them promising candidates for optoelectronics and other advanced technological applications. Concurrently, the pre‐synthesized MOFs provide a surface that is conducive to the nucleation and subsequent growth of PeNCs. Building upon the PeNCs formation pathway within MOFs, the ship‐in‐bottle strategy can be further categorized into four approaches: physical blending, direct transformation, in‐situ deposition, and sequential deposition. The sequential deposition method involves the step‐wise introduction of perovskite precursors (e.g., ionic components, intermediates, and solvent components) into the pre‐synthesized MOFs, thereby inducing perovskite formation within the MOF cavities. A significant challenge associated with PeMOFs synthesis via sequential deposition is the limited control over the shape and size of the resulting PeNCs.

**Figure 5:**
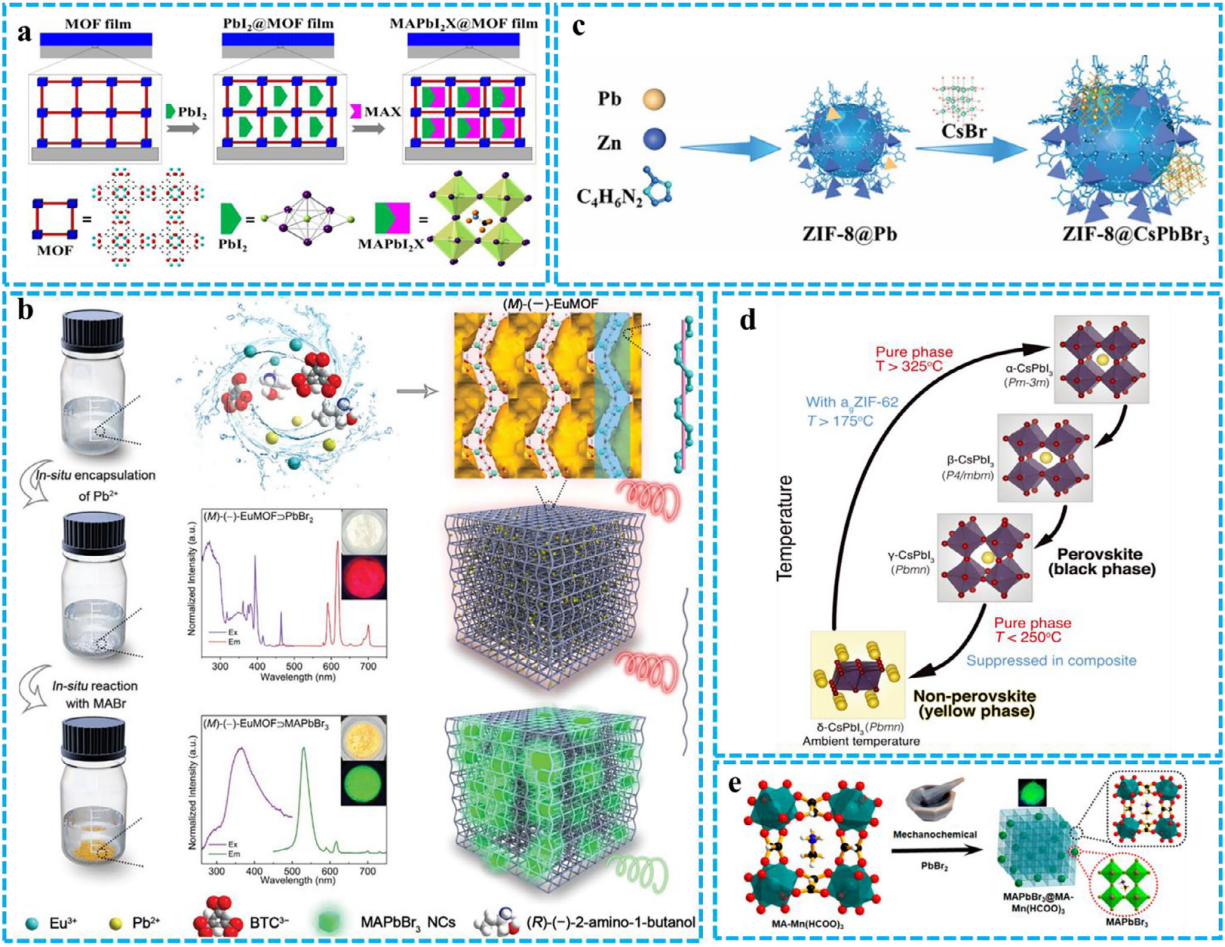
Ship‐in‐bottle synthesis of PeMOFs. a) Sequential deposition method for synthesizing MAPbX_3_@Eu‐MOFs composites. Reproduced with permission.^[^ ^63^ ^]^ Copyright 2016 American Chemical Society, b) In‐situ deposition method for synthesizing EuMOF@MAPbX_3_. Reproduced with permission.^[^ ^64^ ^]^ Copyright 2022 Wiley. c) Direct transformation method for synthesizing CsPbBr_3_@ZIF‐8. Reproduced with permission.^[^ ^65^ ^]^ Copyright 2023 Wiley, d) Liquid phase sintering method for synthesizing (CsPbI_3_)_0.25_(ZIF‐62)_0.75_. Reproduced with permission.^[^ ^66^ ^]^ Copyright 2021 American Association for the Advancement of Science, e) Mechanochemical grinding method for synthesizing PeMOFs. Reproduced with permission.^[^ ^67^ ^]^ Copyright 2020 American Chemical Society.

In 2016, Chen et al. employed a sequential deposition method to grow MAPbI_2_X within the pores of MOFs (**Figure** **5**a).^[^ ^63^ ^]^ By sequentially introducing PbI_2_ and CH_3_NH_3_X precursors, they successfully synthesized ultra‐small MAPbX_3_ NCs within the microporous MOF material HKUST‐1. The resulting MAPbI_2_X NCs exhibited a consistent diameter of 1.5 to 2 nm, closely matching the pore size of HKUST‐1. This work demonstrates a novel approach to controlling the uniform size of PeNCs by leveraging the oriented porous structure of MOFs. The in‐situ deposition method directly forms PeMOFs by mixing pre‐synthesized perovskite NCs with the constituent parts of MOFs, including metal ions and organic linkers. In addition to spontaneous growth, the growth of PeNCs can also be promoted by changing the reaction temperature or introducing poor solvents to facilitate the PeNCs nucleation and growth within the MOFs pores. Zhang et al. synthesized two crystalline enantiomers (right‐hand (P)–(+)–EuMOF and left‐hand (M)–(−)–EuMOF, that is ((P)–(+)/(M)–(−)–EuMOF@MAPbX_3_) by in‐situ deposition method. In the first step, the formation of (P)–(+)/(M)–(−)–EuMOF@PbX_2_ by solvothermal self‐assembly of Eu^3+^, fully deprotonated chiral ligand 1,3,5‐benzenetricarboxylic acid, PbBr_2_, and chiral dopants (S)–(+)‐2‐amino‐1‐butanol and (R)–(−)‐2‐amino‐1‐butanol were involved.^[^ ^64^ ^]^ The second step, on the basis of the formed (P)–(+)/(M)–(−)–EuMOF@PbX_2_, PeNCs were quickly formed by reacting with MAX trigger in an ethanol solution (Figure 5b). The direct conversion method involves mixing perovskite precursors with MOF components to generate PeNCs within the MOFs pores by carefully controlling reaction parameters. Guo et al. developed a direct transformation method for synthesizing ZIF‐8@CsPbBr_3_ NCs. The process entailed combining a Pb source with MOF ligands to form a bimetallic MOF, which was subsequently subjected to a post‐treatment step to convert the Pb into CsPbBr_3_ NCs.^[^ ^65^ ^]^ They were also able to preponderate over the size and boosting crystallization with the help of varying the concentration of metal salts and the doping amount of Pb (Figure 5c). This method, through partial decomposition of the MOF structure, enables the rapid and efficient incorporation of PeNCs into the MOF matrix, resulting in a significant enhancement of its loading capacity. The physical blending method, which includes techniques such as ultrasonication, stirring, ultrasonic dispersion, or vacuum adsorption, aims to achieve a well‐dispersed distribution of PeNCs within the pores of MOFs. Recently, Wang et al. introduced an innovative approach to PeMOF preparation by integrating PeNCs with glassy MOF materials.^[^ ^66^ ^]^ They proposed a liquid‐phase sintering technique to fuse PeNCs within the MOF glass matrix (Figure 5d). Rambabu et al. reported a solvent‐free green synthesis strategy for MAPbBr_3_ NCs within PeMOFs [MA–M(HCOO)_3_] [M = Mn and Co; MA. In this approach, the PeMOFs serves as both a source and a directing agent for the MA cation, facilitating the formation and stabilization of the hybrid PeMOFs and the subsequent generation of PeNCs.^[^ ^67^ ^]^ The composite material was synthesized solely through mechanochemical techniques, involving the mechanical milling of the PeMOFs with PbBr_2_, without the need for additional reagents (Figure 5e).

This method effectively optimizes and enhances both the chemical purity and crystalline quality of the PeNCs. The sequential deposition method is relatively complex, requiring multiple steps, and can be challenging to precisely control PeNCs size and shape. The in‐situ deposition method, while simple to implement and offering a controllable deposition process with a high recovery rate, may suffer from limited diffusion of perovskite precursors within the MOFs pores, potentially hindering nucleation and growth efficiency. The direct conversion method effectively circumvents diffusion limitations, enabling precise control over PeNCs size and morphology. Finally, the physical blending method, favored for its operational simplicity and avoidance of complex chemical reactions, is well‐suited for rapid preparation. However, it necessitates MOFs with sufficiently large pores to accommodate the entry of pre‐formed PeNCs. Based on various preparation methods, a new approach combines physical vapor deposition with other techniques to prepare PeMOF composites in a specific vacuum environment, enabling precise atomic–level control for unique structures and properties. Moreover, from an environmental perspective, developing green and sustainable synthesis methods is crucial. Using renewable resources, avoiding toxic substances, and reducing energy and waste are key to green PeMOF composite synthesis, though it remains a significant challenge today.

#### 3.2.2 Bottle‐Around‐Ship

**Figure 6:**
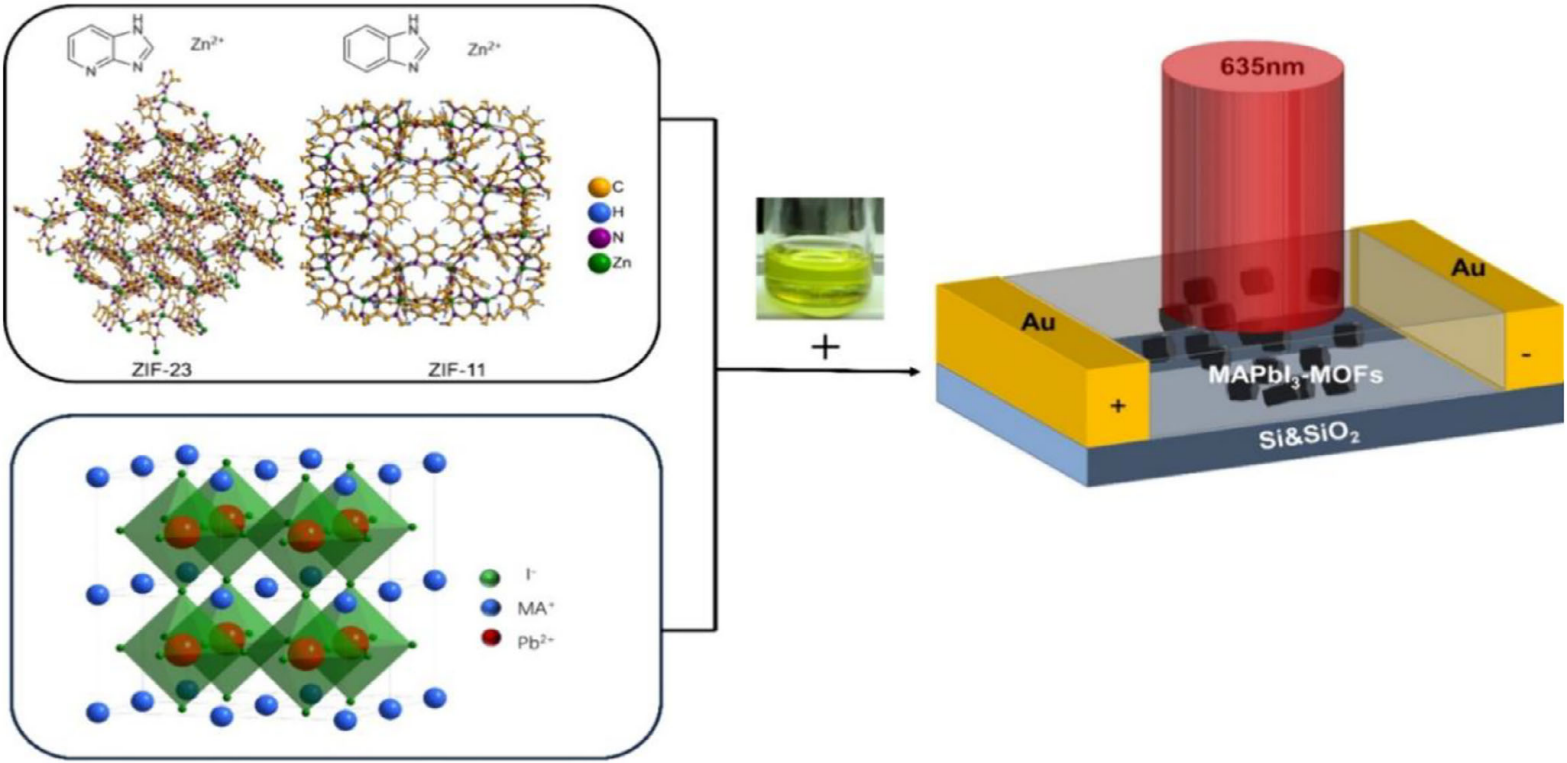
Bottle‐around‐ship synthesis of PeMOFs. Reproduced with permission.^[^ ^68^ ^]^ Copyright 2024 American Chemical Society.

In the “ship‐in‐bottle” approach, PeNCs are formed directly within the pores of MOFs. Conversely, the “bottle‐around‐ship” approach involves encapsulating pre‐synthesized PeNCs within a MOF shell. A key advantage of the latter method is that the size of the PeNCs is not constrained by the MOFs pore size, allowing for the incorporation of larger or more precisely controlled PeNCs. Moreover, this approach mitigates the formation of unwanted PeNCs on the MOFs' external surface, as only the internal pore environment facilitates well‐defined PeNCs nucleation and growth. This is particularly beneficial for the synthesis of PeMOFs, as it leads to a uniform distribution of PeNCs within the MOF framework, thereby enhancing its optical and stability properties. However, this method is highly sensitive to the assembly process, requiring careful control to prevent the formation of excess PeNCs outside the MOF structure. For example, Guan et al. prepared MAPbI_3_–MOFs using a similar “out‐of‐vessel” method. They synthesized ZIF‐11 and ZIF‐23 via a solvothermal route and then dispersed these ZIFs in a mixture of ethanol and acetonitrile (**Figure** **6**).^[^ ^68^ ^]^

While Bottle‐around‐ship method enhances the flexibility and freedom in the synthesis of PeMOFs, it introduces a significant challenge: the assembly and growth of MOFs typically involve polar solvents such as water, ethanol, and N, N‐dimethylformamide (DMF). Furthermore, the synthesis of some MOFs materials requires harsh conditions, including high temperatures and acidic or basic environments, which can lead to the decomposition of the perovskite component and further compromise the overall stability of the PeMOF. To mitigate these issues, we propose the following strategies: 1) employing more robust PeNCs; 2) utilizing MOFs that can be synthesized under milder conditions as the host material; and 3) carefully selecting solvents with appropriate, moderate polarity.

In summary, both the ship‐in‐bottle and bottle‐around‐ship methods offer viable routes for encapsulating PeNCs within MOFs matrices. However, it is important to acknowledge the limitations of each approach. The ship‐in‐bottle method requires careful consideration of diffusion limitations and precise control over the size, position, and morphology of the encapsulated PeNCs. Conversely, while the bottle‐around‐ship technique provides greater synthetic flexibility and facilitates the preparation of high‐purity PeMOFs composites, its long‐term stability can be a concern. Thus, it is necessary to explore new synthetic methods. For example, combining physical vapor deposition technology with other methods can synthesize MOFs and perovskites in a specific vacuum environment. This can achieve precise atomic–level control and produce composite materials with unique structures and properties. Moreover, considering environmental friendliness, it's important to develop green and sustainable synthesis methods. Using renewable resources as raw materials, avoiding toxic chemicals and solvents, and reducing energy consumption and waste emissions can achieve green synthesis of MOF–perovskite composite materials.

## 4 The Role of MOFs in Perovskites

**Figure 7:**
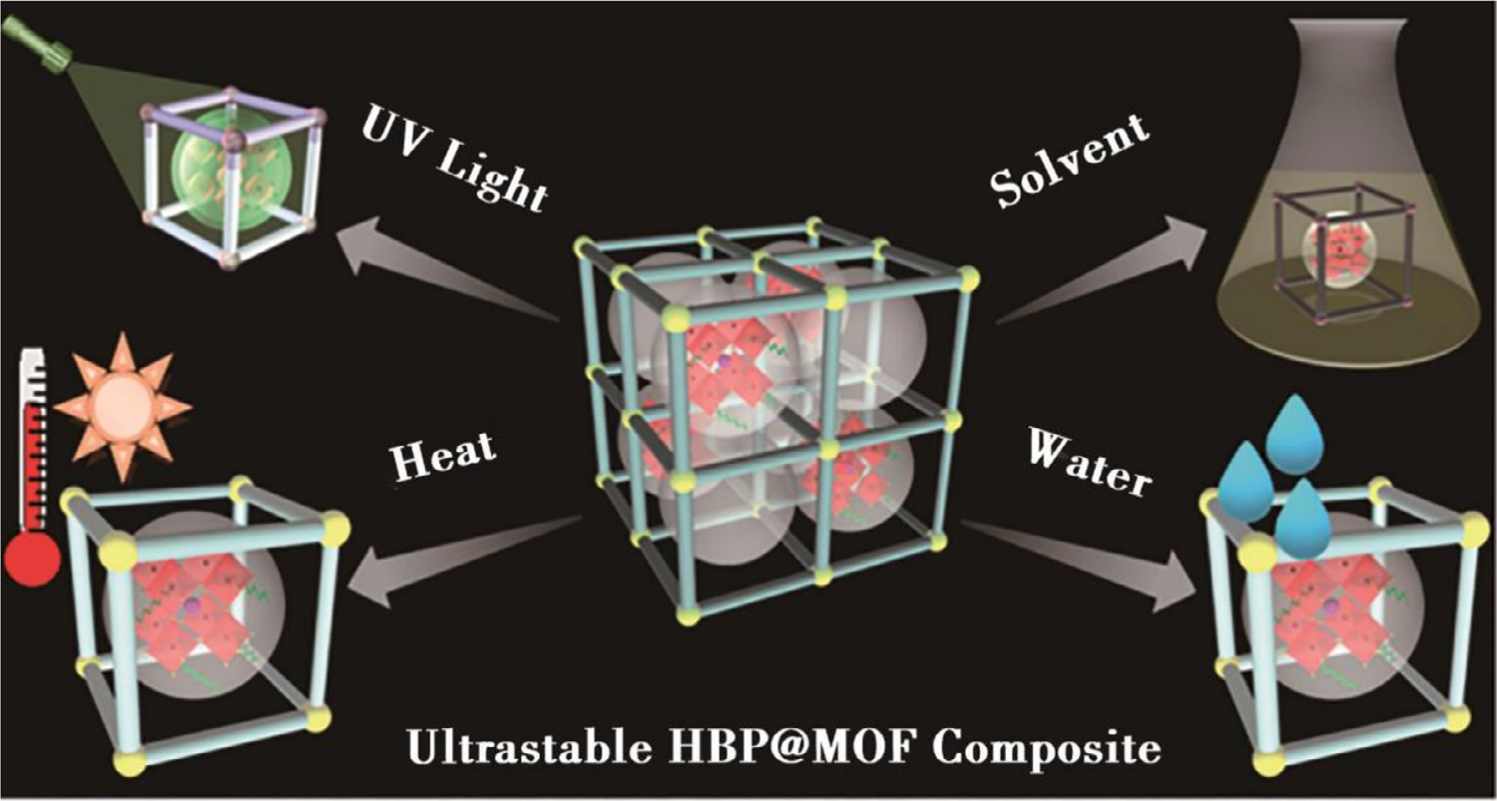
Schematic diagram of MOFs increasing perovskite stability. Reproduced with permission.^[^ ^69^ ^]^ Copyright 2024 Changchun Institute of Optics, Fine Mechanics and Physics.

Although perovskite materials (particularly halide perovskites such as MAPbI_3_ and CsPbBr_3_) exhibit outstanding optoelectronic properties, their inherent defects severely hinder practical applications. They suffer from poor water stability, poor thermal stability, poor photostability, and lead ion toxicity. MOFs are highly valued for their exceptional thermal and chemical stability, unique porous structures, and the ability to tailor their physical and chemical properties. These attributes make them promising candidates for enhancing the performance and stability of perovskite devices. In this emerging field, a range of intriguing functionalities of MOFs have been identified and effectively utilized, including their roles as porous templates to control perovskite layer crystallization, defect mitigators, light scattering enhancers, and UV filters (**Figure** **7**). Despite the relatively nascent stage of the PeMOFs composite field, several fundamental questions have arisen: How does charge transfer occur between PeNCs and MOFs, How does encapsulation within MOFs preserve the physical and chemical integrity of PeNCs? Researchers synthesizing PeMOFs composites continue to face challenges related to optimizing material properties and stability. The following sections of this manuscript will discuss and analyze the active roles of MOFs in relevant PeNCs applications.

### 4.1 Confinement of PeNCs Assembled in MOFs

**Figure 8:**
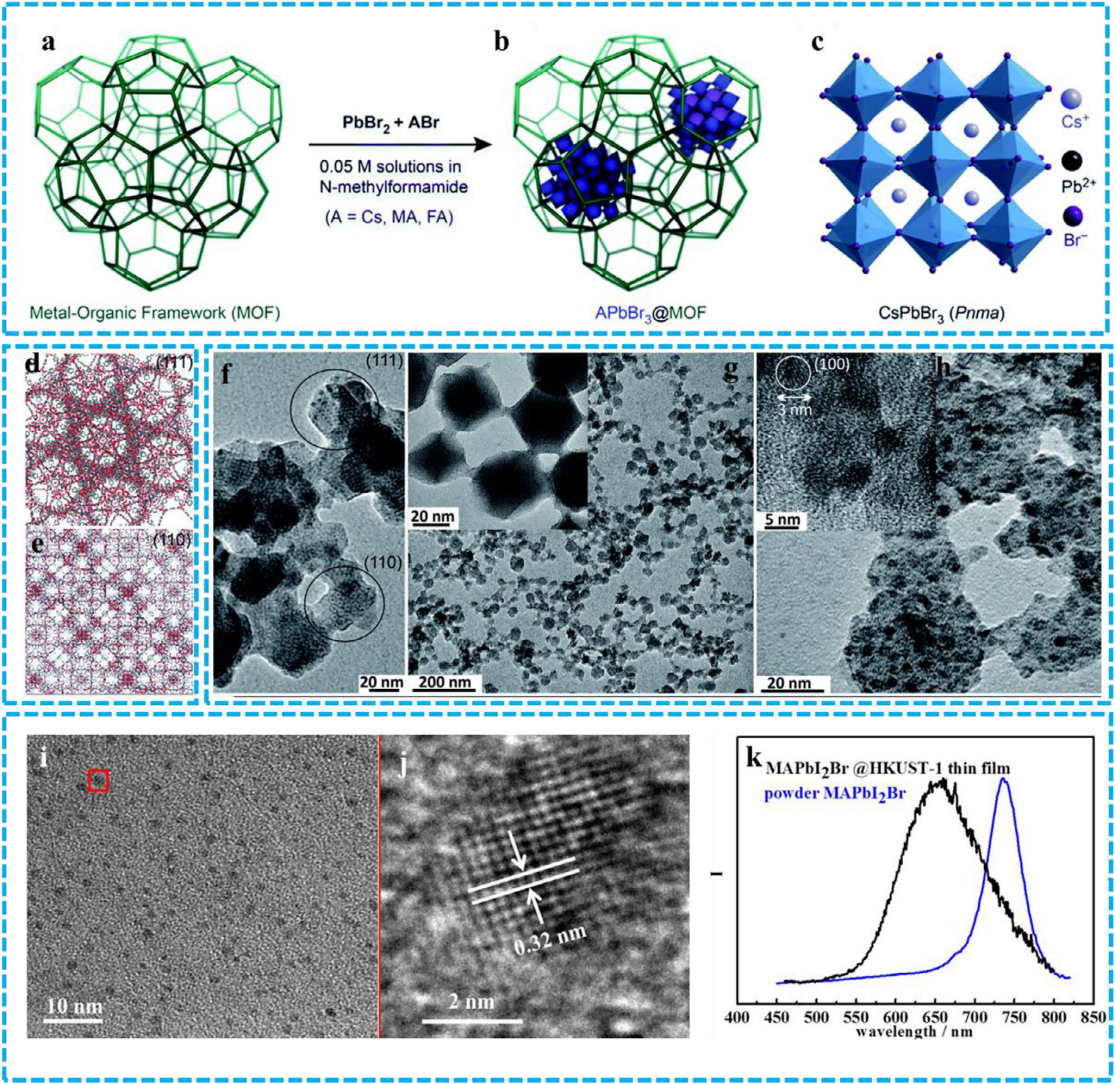
a) An illustration depicting the 3D how porous the MOFs are prior to the encapsulation of PeNCs, b) an image showing the MOFs post‐encapsulation, with the inclusions symbolizing the PeNCs entrapped within the pores, and c) crystal structure for g‐orthorhombic CsPbBr_3_ (Pnma) d) a depiction of the (111) plane within Cr–MIL–101, e) a visualization of the (110) plane within Cr–MIL–101, f,g) TEM images of Cr–MIL–101 (≈25 nm in size), h) TEM images of CsPbBr_3_@Cr–MIL–101 (≈25 nm in size). Reproduced with permission.^[^ ^70^ ^]^ Copyright 2021 Royal Society of Chemistry. i) TEM image, j) HRTEM with lattice spacing, and k). PL spectrum of MAPbI_2_Br@HKUST‐1 thin film. Reproduced with permission.^[^ ^63^ ^]^ Copyright 2016 American Chemical Society.

PeNCs can be grown in situ within the framework of MOFs, resulting in the formation of a homogeneously distributed heterostructure. The porous architecture of the MOF acts as a well‐defined matrix, effectively constraining the growth of PeNCs and promoting the formation of extremely fine NCs. Furthermore, the robust MOF framework isolates the PeNCs, significantly reducing the tendency for crystal aggregation. For example, Loredana et al. synthesized APbBr_3_@MOFs using the ship‐in‐a‐bottle technique (**Figure** **8**a–c).^[^ ^70^ ^]^ Top‐view illustrations of the (111) and (110) planes of Cr_3_O(OH)(H_2_O)_2_(terephthalate)_3_ (Cr–MIL) are presented in Figure 8d–f, confirming the successful fabrication of the Cr–MIL structure. Transmission electron microscopy (TEM) images of PeNCs encapsulated within the MOF reveal a particle size of ≈3 nm (Figure 8g,h), which is consistent with the cage dimensions of the MOFs (2.9 and 3.4 nm). Chen et al. achieved highly dispersed MAPbI_2_Br_1_ NCs smaller than 5 nm within the oriented MOFs matrix (Figure 8i,j).^[^ ^63^ ^]^ This confinement effect led to an 80 nm blue‐shift in the PL emission peak of MAPbI_2_Br_1_ NCs compared to bulk MAPbI_2_Br_1_ (Figure 8k), demonstrating a method for tuning emission colors. Although MOFs have a regular pore structure that can provide confined space for the growth of PeNCs, thereby achieving precise control of their size, the growth of PeNCs in MOFs pores may encounter diffusion limitations, which can affect the growth and performance of perovskites. Therefore, in the following practical operations, controlling the size and shape of PeNCs remains challenging, especially in large‐scale production.

### 4.2 MOFs Shield the PeNCs from Oxygen and Moisture

Previous research indicates that PeNCs are susceptible to degradation from oxygen and moisture, leading to ligand detachment and emission quenching.^[^ ^71^, ^72^ ^]^ To address this, incorporating MOFs as a protective shell for perovskite quantum dots offers a promising strategy. The high porosity and large specific surface area of MOFs effectively shield the perovskite core from environmental degradation, eliminating the need for stabilizing solvents. Furthermore, the abundant functional groups within MOFs facilitate strong binding with perovskite quantum dots, ensuring their stable anchoring within the MOF pores and preventing aggregation. This isolation minimizes degradation caused by external stimuli. In addition, the porous structure and inherent hydrophobicity of certain MOFs can mitigate heat transfer to the PeNCs and impede water intrusion. These combined attributes contribute to the enhanced environmental stability observed in PeMOF composites.

**Figure 9:**
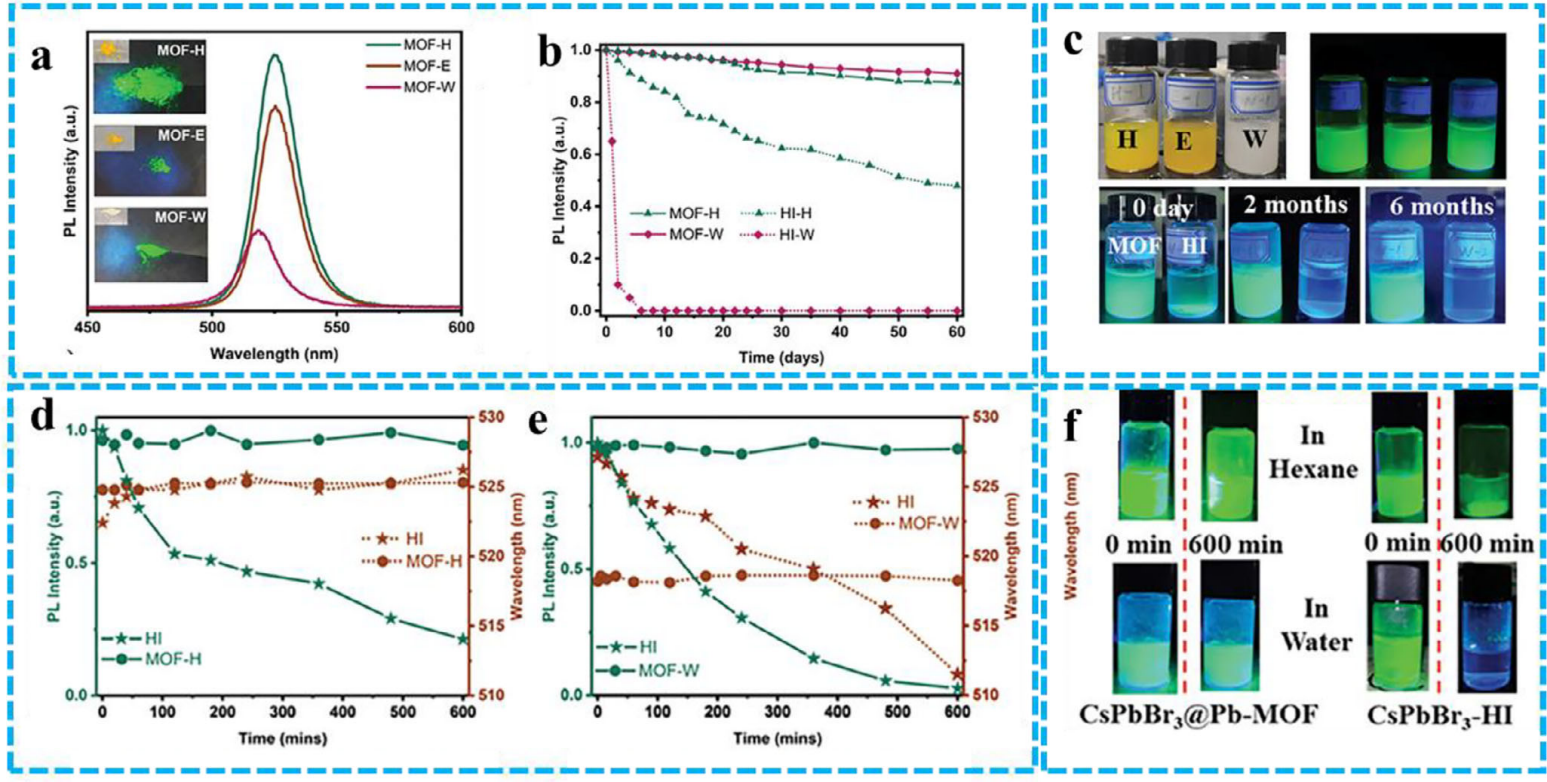
a) PL intensity measurements of CsPbBr_3_@Pb‐MOFs composites in hexane, ethanol, and water are presented, with the accompanying inset figures displaying the powder samples under both ambient illumination and 365 nm UV light. b) The optical images of the samples, arranged from left to right in hexane, ethanol, and water, are shown under both ambient lighting and 365 nm UV illumination (top row). Additionally, the images display CsPbBr_3_@Pb‐MOFs (on the left) and CsPbBr_3_‐HI (on the right) in water under 365 nm UV light at intervals of 0 days, 2 and 6 months (bottom row). c) This section examines the long‐term stability of CsPbBr_3_@Pb‐MOFs and CsPbBr_3_‐HI in both hexane and water. The resistance to UV radiation for CsPbBr_3_@Pb‐MOFs composites and CsPbBr_3_‐HI suspended in d) hexane and e) water. (f) PL images before and after 600 min of UV exposure for both CsPbBr_3_@Pb‐MOFs composites (left side) and CsPbBr_3_‐HI (right side) in hexane and water. Reproduced with permission.^[^ ^73^ ^]^ Copyright 2024 Wiley.

Wang et al. successfully synthesized CsPbBr_3_@Pb‐MOF composites and investigated the optical properties of the resulting powders after centrifugation in water (MOFs‐W) and ethanol (MOFs‐E).^[^ ^73^ ^]^ Their findings revealed that, despite the presence of Pb‐MOFs, the optical performance of the composites still exhibited a decrease (**Figure** **9**a). However, in comparison to unencapsulated CsPbBr_3_, the CsPbBr_3_@Pb‐MOF composites demonstrated significantly enhanced long‐term stability when dispersed in both hexane and water. Notably, after two months of immersion in water, the composites retained over 90% of their initial PL intensity (Figure 9b,c). Furthermore, under 365 nm UV irradiation for 600 min, the PL intensity and central wavelength of the CsPbBr_3_@Pb‐MOF composites in cyclohexane solution remained stable (Figure 9d). In contrast, traditional CsPbBr_3_‐Hot Injection (HI), experienced surface ligand removal under UV irradiation, leading to nanoparticle aggregation and an 80% reduction in PL intensity. Figure 9e illustrates the PL intensity changes of the CsPbBr_3_@Pb‐MOF composites under continuous 365 nm UV exposure in an aqueous environment. The composites exhibited superior UV resistance compared to bare CsPbBr_3_, maintaining stable PL intensity even in water. Visual confirmation of the stability under various conditions is provided in Figure 9f. Encapsulation, as demonstrated here, hold great promise as a strategy for enhancing the stability of PeNCs in future research.

### 4.3 The Enhancement of the Catalytic Performance of PeNCs by MOFs

**Figure 10:**
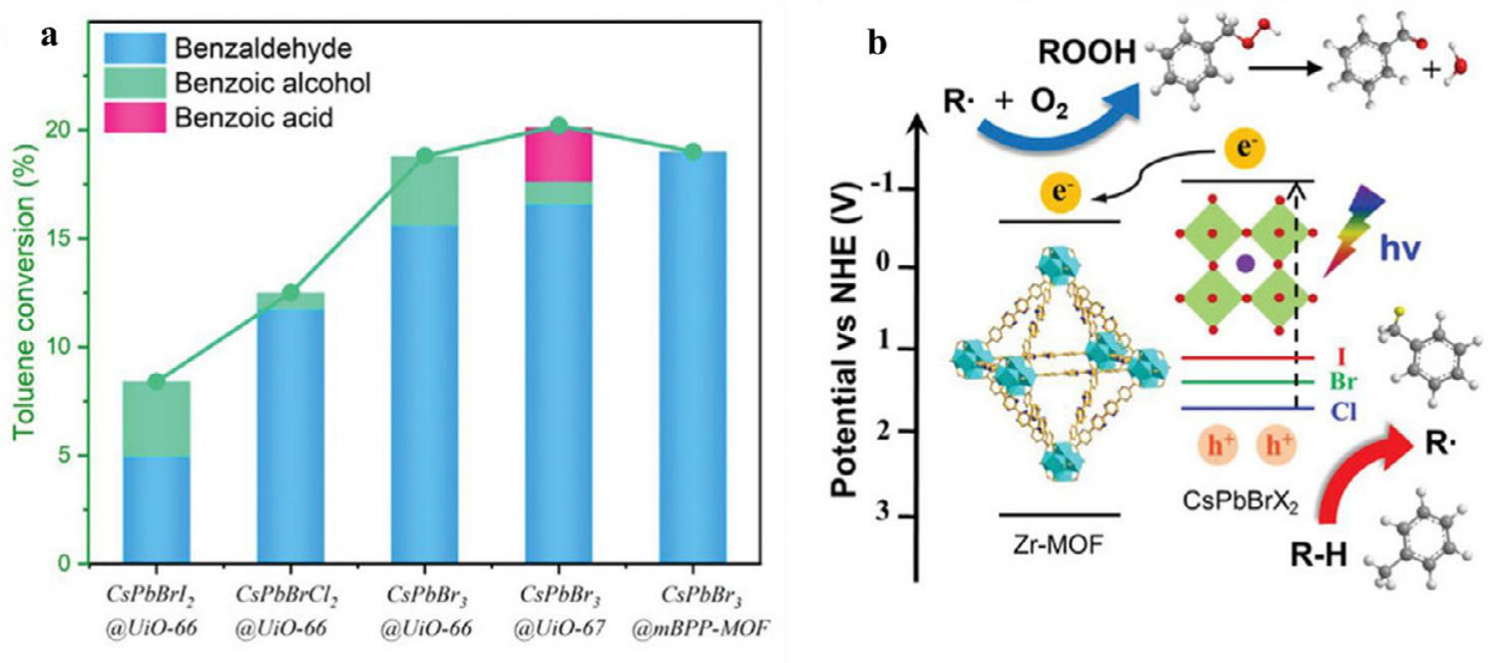
a) The toluene conversion rate and product yield of PeMOF. b) The catalytic mechanism diagram of PeMOFs. Reproduced with permission. Reproduced with permission.^[^ ^76^ ^]^ Copyright 2024 Wiley.

MOFs exhibit high surface areas and abundant porous structures, providing numerous active sites for perovskite nanocrystals (PeNCs) and consequently increasing the adsorption and reaction opportunities for reactants.^[^ ^74^ ^]^ Furthermore, the porous nature of MOFs facilitates the diffusion of both reactants and products, leading to enhanced catalytic efficiency. The formation of heterojunctions between MOFs and PeNCs can effectively separate photogenerated carriers, improving charge transport efficiency and boosting photocatalytic performance.^[^ ^75^ ^]^ For instance, Liu et al. synthesized CsPbBr_3_@mBPP‐MOF using the ship‐in‐bottle method.^[^ ^76^ ^]^ A comparative analysis of PeNCs, MOFs, and PeNCs@MOF composites revealed that neither individual CsPbBr_3_ NCs nor pristine MOFs produced oxidative products due to significant electron‐hole recombination. Notably, all PeNCs@MOF hybrids displayed catalytic activity, leading to the successful oxidation of C─H bonds. (**Figure** **10**a; **Table** **1**). Upon irradiation with visible light (395 nm), photogenerated electron‐hole pairs are created within the conduction band minimum (CBM) and valence band maximum (VBM) of the PeNCs. In the heterojunctions formed within the PeNCs@MOF composite, the staggered energy bands facilitate the efficient migration of electrons from the CBM of PeNCs to the LUMO level of the MOF, leaving holes in the VBM (Figure 10b). This electron transfer significantly reduces photogenerated carrier recombination, thereby enhancing the overall catalytic performance. This observation highlights that electron transfer from PeNCs to MOFs can enhance the catalytic performance of PeNCs to a certain extent, potentially offering valuable insights into the practical application of PeNCs in photocatalysis. Moreover, leveraging the electron flow from PeNCs to MOFs has yielded promising results in various PeNCs‐based optoelectronic devices. This approach holds great promise for future research on PeNCs.

**Table 1:** CsPbX_3_@MOFs as an Efficient Photocatalyst for Toluene Oxidation under 395 nm Light Irradiation.^[^ ^76^ ^]^ Copyright 2024 Wiley.

Catalyst	Conversion	Selectivity (%)		
		benzaldehyde	benzyl alcohol	benzoic acid
CsPbBr_3_ NCs	0	0	0	0
UiO‐67‐bpy	0	0	0	0
mBPP‐MOF	0	0	0	0
CsPbBrI_2_@ UiO‐66	8.4	58.9	41.1	0
CsPbBrCl_2_@ UiO‐66	12.5	93.8	6.2	0
CsPbBr_3_@ UiO‐66	18.8	82.9	17.1	0
CsPbBr_3_@ UiO‐67‐bpy	20.2	82.3	5.2	12.5
CsPbBr_3_@ mBPP‐MOF	55.3	33.3	0	66.4

### 4.4 MOFs Provide Ion Exchange Pathways for Perovskites

**Figure 11:**
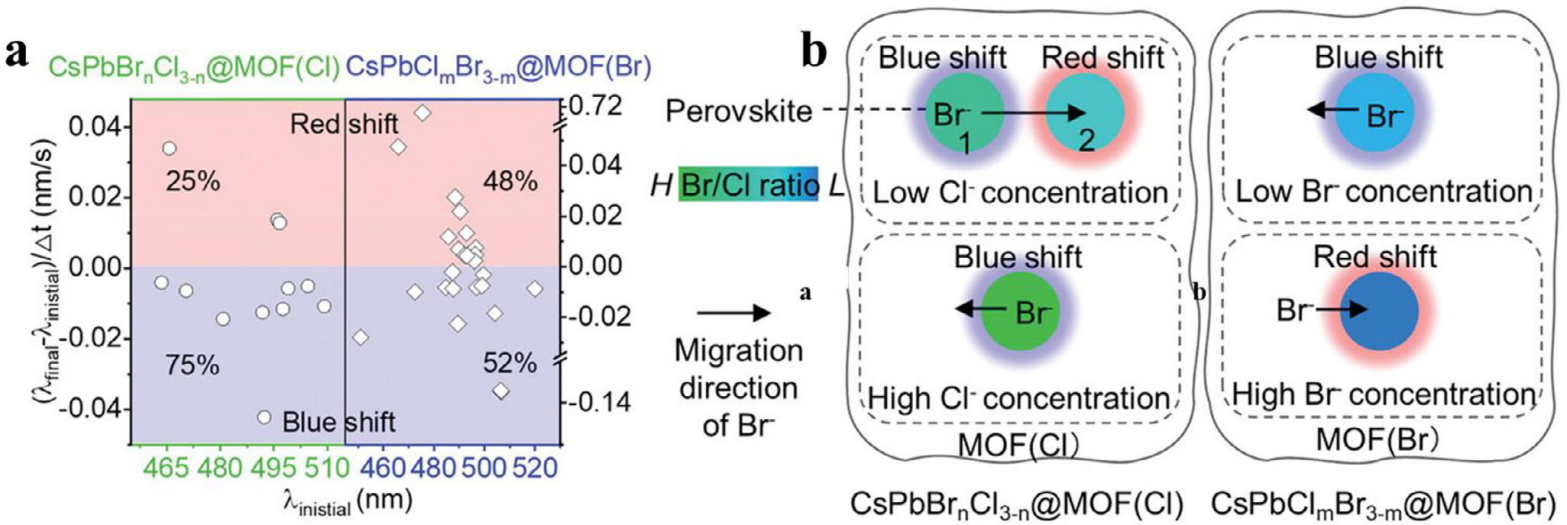
a) Distribution of the average wavelength shift ((λ_final_ − λ_initial_)/Δt) as a function of the initial peak wavelength position. b) Schematic diagram of Br^−^ migration for CsPbBr_n_Cl_3‐n_@MOF(Cl) (left) and CsPbCl_m_Br_3‐m_@MOF(Br) (right). Reproduced with permission.^[^ ^77^ ^]^ Copyright 2024 Wiley.

MOFs not only protect perovskites, improve their catalytic performance, and regulate their morphology, but also influence the composition and thereby the properties of perovskites. Vacha and his team employed a series of CsPbX_3_@MOF(X) (X = Br or/and Cl) composites to systematically investigate their nanoscale photoluminescence properties.^[^ ^77^ ^]^ By real‐time tracking of the crystallization kinetics of CsPbX_3_ NCs within MOF(X), they uncovered the key regulatory role of MOF(X) in perovskite composition. The study revealed a non‐monotonic matrix‐halide dependence in the spectral evolution of hybrid halide perovskites, pointing to dynamic modulation by halide ion migration (**Figure** **11**a,b). More significantly, it was confirmed that collective halide ion migration could lead to spatially synchronized photoluminescence blinking. These findings offer a fresh perspective on the fundamental photophysical properties of perovskite‐MOF composite systems. They are expected to promote in‐depth research on ion migration in mixed–halide solids and help reveal limitations of perovskite– based optoelectronic devices.

## 5 Applications for PeMOFs

Following extensive research into the synthesis, design, and characteristics of PeNCs@MOFs composites, current efforts are increasingly directed towards enhancing the stability of PeNCs to enable their wider practical application. The subsequent section highlights recent progress in PeNCs@MOFs hybrid technology, demonstrating its potential across diverse fields including sensing, photocatalytic degradation of organic pollutants, Anti‐counterfeiting and encryption, solar energy conversion, and light‐emitting diodes (LED) (**Table** **2**).

**Table 2:** Applications of PeNCs@MOFs.

Perovskite	MOF	Synthesis method	Application	Refs.
CsPbBr_3_	UiO‐67	Sequential Deposition	White LED	[78]
CsPbX_3_	HP‐UiO‐66	Physical mixing method		[79]
CsPbClBr_2_	ZIF‐8	Bottle‐around‐ship		[80]
(PhBA)_2_Cs_3_Pb_4_Br_3_	MOF‐5	Microwave‐assisted		[81]
CH_3_NH_3_PbBr_3_	Bio‐MOF‐1	Bottle‐around‐ship		[82]
MAPbBr_3_, CsPbBr_3_	Pb‐BTC	Direct conversion method	Green LED	[24]
CsPb(BrCl)_3_	Pb‐BTC	Direct conversion method	Blue LED	[83]
MAPbI_3_	UiO‐66‐(SH_2_)	Physical mixing method	Solar Cells	[84]
MAPbI_3_	Zn‐MOF	Physical mixing method		[85]
FAPbI_3_	Ni‐MOF‐74/ Zn‐MOF‐74	Physical mixing method		[27]
CsPbBr_3_	ZIF‐8	Direct conversion method	Photocatalytic	[65]
MAPbI_3_	PCN–221(Fe_x_)	Sequential deposition method		[75]
CsPbI_3_	PCN‐222	Sequential deposition method		[25]
CsPbBr_3_	Pb‐MOF	Direct conversion method		[86]
CH_3_NH_3_Br	ZIF‐90	In‐situ deposition method	Sensors	[87]
CsPbBr_3_	Pb‐MOF	Direct conversion method		[88]
CsPbBr_3_	Cu‐MOF	Physical mixing method		[89]
CsPbBr_3_	MOF‐5	In‐situ deposition method		[90]
MAPbBr_3_	Pb‐MOF	Direct conversion method	Anti‐counterfeiting and encryption	[26]
MAPbBr_3_	Eu‐MOF	In‐situ deposition method		[64]
MAPbBr_3_	Pb/Eu‐MOF	Direct conversion method		[91]
MAPbI_3_	ZIF‐23/ ZIF‐11	Bottle‐around‐ship	Photoelectric detector	[68]
MAPbI_3_	ZIF‐8	Bottle‐around‐ship		[92]
MAPbBr_3_	bMOF	Sequential Deposition Method	scintillators	[93]
MAPbBr_3_	UiO‐66	In‐situ deposition method		[94]

### 5.1 Light‐Emitting Diodes (LED)

#### 5.1.1 PeMOFs for Phosphor‐Converted (pc) LEDs

**Figure 12:**
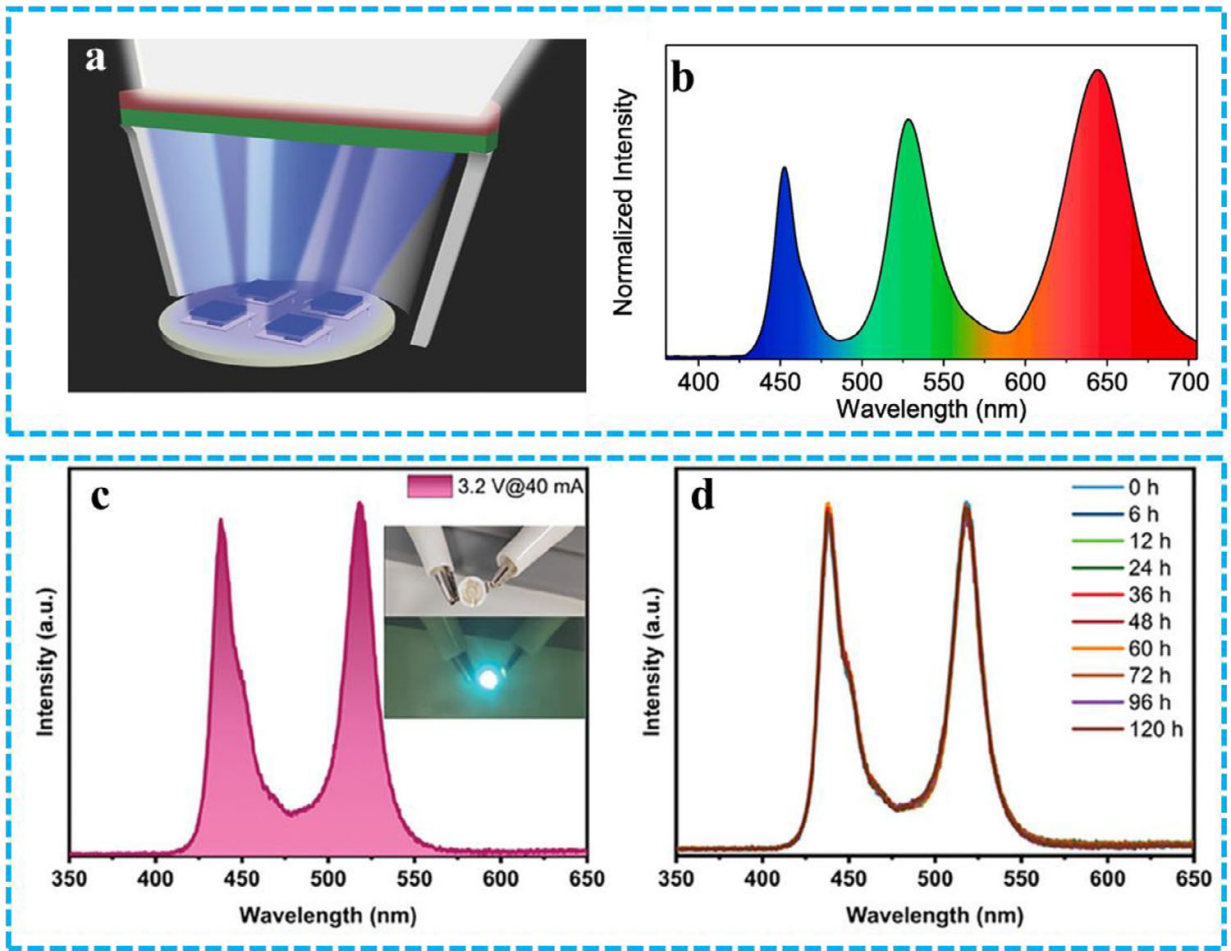
a) Diagram of the WLEDs combining blue LED chips with PeNCs@MOFs composites. Reproduced with permission.^[^ ^97^ ^]^ Copyright 2016 Wiley. b) The emission spectrum of WLEDs based on CsPbBr_3_@Pb‐MOFs. Reproduced with permission.^[^ ^98^ ^]^ Copyright 2021 Elsevier. c) Green LEDs based on PeNCs@MOFs composites. d) Long‐term stability of WLEDs immersed in water. Reproduced with permission.^[^ ^73^ ^]^ Copyright 2024 Wiley.

Phosphor‐converted (pc) LEDs, widely used for indoor white illumination and commercial displays, consist of a blue LED die coated with one or more layers of phosphorescent materials. In this configuration, a portion of the blue light emitted by the LED is absorbed by the phosphor and re‐emitted at longer wavelengths as visible light, resulting in the generation of white light. While this technology is prevalent, challenges remain, particularly in sourcing phosphor materials capable of emitting a diverse range of colors. Balancing the emitted wavelengths to achieve the desired white light characteristics (warm or cool) can also be complex. A key advantage of pc‐LEDs is the ability to mix different emitting pigments, allowing for precise control over the red, green, and blue light ratio, as illustrated in **Figure** **12**a.^[^ ^95^ ^]^ Guan et al. reported a luminescent CsPbX_3_@HP‐UIO‐66 (X = Br or I) nanostructure exhibiting both intense luminescence and robust resistance to environmental humidity, UV radiation, and anion exchange.^[^ ^96^ ^]^ This nanostructure maintained stable spectral output over 72 h of continuous operation. By integrating traditional blue InGaN LED chips with green‐emitting CsPbBr_2_I_1_@HP‐UiO‐66 and red‐emitting CsPbI_3_@HP‐UiO‐66 composites, they successfully created a white LED prototype achieving a Rec. 2020 color gamut coverage of 93% and an NTSC coverage of 125% (Figure 12b).

A key challenge in phosphor‐converted LEDs (pc‐LEDs) is achieving stable brightness and precisely controlled emission color from phosphor materials under consistent blue or UV LED illumination. As previously noted, prolonged light exposure can degrade PeNCs unless protective measures are implemented. This degradation can be exacerbated by humidity, and UV irradiation can induce PeNCs aggregation, leading to a reduction in emission intensity and a shift in the emitted color.^[^ ^97^ ^]^ These issues significantly hinder the development of high‐performance pc‐LEDs. To address this, Wang et al. synthesized CsPbBr_3_@Pb‐MOFs and utilized them as a backlight with a commercial 445 nm InGaN LED to create a green light source (Figure 12c).^[^ ^73^ ^]^ The emission performance of unencapsulated LEDs was then evaluated after immersion in water for 120 h (Figure 12d). The results demonstrated that the MOF shell effectively protected the CsPbBr_3_ nanocrystals, as the fabricated LEDs maintained favorable PL characteristics even after prolonged water exposure. While PeMOFs interlayers have shown promise in LED fabrication, the external quantum efficiency (EQE) of these devices remains a limitation. Consequently, researchers are also investigating the application of PeMOFs in direct‐conversion LEDs.

#### 5.1.2 PeMOFs for Direct Conversion LED

PeMOFs can be utilized as the emissive layer in LEDs, where electroluminescence (EL) arises from the direct recombination of electrons and holes injected from selective electrodes within the PeMOFs layer. During LED operation, the application of an external voltage leads to the injection of holes and electrons into the PeMOFs emissive layer from the anode and cathode, respectively. Selective electrodes facilitate this injection process by preferentially allowing the passage of specific charge carriers (electrons or holes). The careful design of these selective electrodes is paramount for achieving high luminous efficiency and stability in LEDs. This is because well‐designed electrodes promote a balanced injection of electrons and holes, thereby optimizing the charge carrier recombination rate. Under the influence of the external electric field, injected electrons and holes migrate towards each other within the PeMOFs layer. The electron and hole mobility within the PeMOFs significantly impacts the efficiency of charge transport. The conductivity of PeMOFs, and consequently their charge transport characteristics, can be tuned by modulating the electronic structure of their metal nodes and organic ligands.

**Figure 13:**
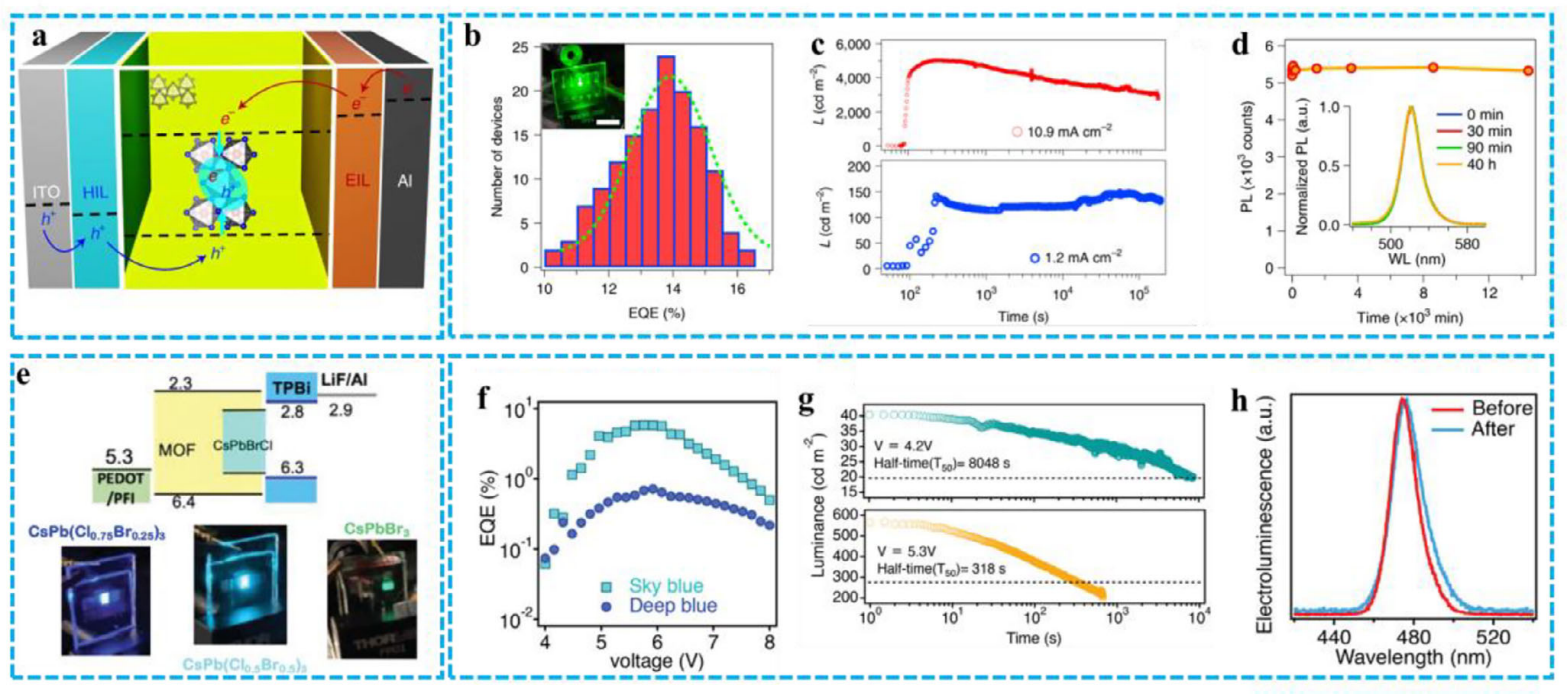
a) A diagram illustrating the structure of LEDs. b) A histogram displaying the external quantum efficiency (EQE) data, with an inset photograph showing the operation of PeMOFs‐based LEDs. c) The normalized photoluminescence (PL) spectrum (inset) and the variation of PL optimum strength over time for the MA‐PeMOF emission layer. d) The normalized electroluminescence (EL) spectra of PeMOFs‐based LEDs, plotted against the duration of operation. Reproduced with permission.^[^ ^24^ ^]^ Copyright 2021 Springer Nature. e) The energy level alignment diagram (top) and a photograph (bottom) of the PeMOFs‐based LEDs. f) The EQE values for PeMOFs‐based LEDs. g) A comparison of PL intensity for sky‐blue, deep‐blue, and green PeMOFs thin films. h) The stability assessment of sky‐blue LEDs. Reproduced with permission.^[^ ^83^ ^]^ Copyright 2022 Wiley.

In a direct‐conversion LED, the PeMOF material is strategically positioned between the electron‐ and hole‐injection electrodes to facilitate electroluminescence. Applying an external voltage causes the anode (hole‐injection electrode) to inject holes and the cathode (electron‐injection electrode) to inject electrons (**Figure** **13**a).^[^ ^24^ ^]^ The PeMOF LED exhibits an average external quantum efficiency of approximately 14%, defined as the ratio of radiated photons to injected electrons (Figure 13b). Although most reported perovskite LEDs degrade within 20 min of continuous operation, Figure 13c demonstrates that the PeMOF device exhibits sustained stability over 20 h under low injection current. Furthermore, linear organic nanocrystals are incorporated into the PeMOF structure of the LED. Figure 13d presents the emission intensity of the MA‐PeMOF layer and its normalized PL spectra at different operating times. Notably, even when the LED's brightness decreases to 50% of its initial value, the PL peak position and intensity remain constant, indicating that nanocrystal aggregation does not occur during LED aging. A blue PeMOF LED exhibiting these results is shown in Figure 13e. It generates a deep blue and sky‐blue light. As illustrated in Figure 13f, the blue LED's external quantum efficiency can reach 5.6%. Interestingly, the lifespan results extended even further at greater injection currents by incorporating all‐inorganic PeNCs, like CsPbBr_3_, inside MOFs. (Figure 13g). The PL spectra before and after high bias stress in Figure 13h demonstrates that the Cs‐PeMOF device's PL optimum location is nearly constant, with a slight trace pointing downward. This indicates that most nanocrystals do not undergo significant phase segregation. It is suggested that PeMOF offers enhanced thermal stability, enabling it to effectively manage the Joule heat generated by constant current injection, making it well‐suited for use in direct conversion LEDs. Overall, PeMOF holds great potential in LED applications. Although its development is still in its infancy, a variety of novel physical properties may be realized by assembling various types of PeNCs into new MOF structures.^[^ ^83^ ^]^

### 5.2 Solar Cells

Perovskite solar cells (PSCs) offer a green and efficient route for converting solar energy into electrical energy, establishing them as a leading photovoltaic technology. While PeNCs are widely utilized in solar cells, the long‐term stability of PSCs remains a significant challenge.^[^ ^98^ ^]^ PeMOF composites have emerged as a promising solution to address this issue. MOFs, characterized by their high specific surface area and open‐channel structure, facilitate ion and electron transport. Furthermore, the strong coordination between metal ions and linkers in MOFs typically imparts excellent moisture and chemical stability. Incorporating pre‐synthesized MOF crystal precursor solutions into PeNCs allows MOF crystals to be embedded at the perovskite film grain boundaries. These embedded MOFs can then act as a mesoporous scaffold, effectively regulating the PeNCs material. MOFs play a significant role in controlling perovskite crystallization, passivating defects, and enhancing charge carrier mobility. When used as additives in PSCs, MOFs can also effectively passivate interfacial defect states, leading to improved optoelectronic performance and device stability.

**Figure 14:**
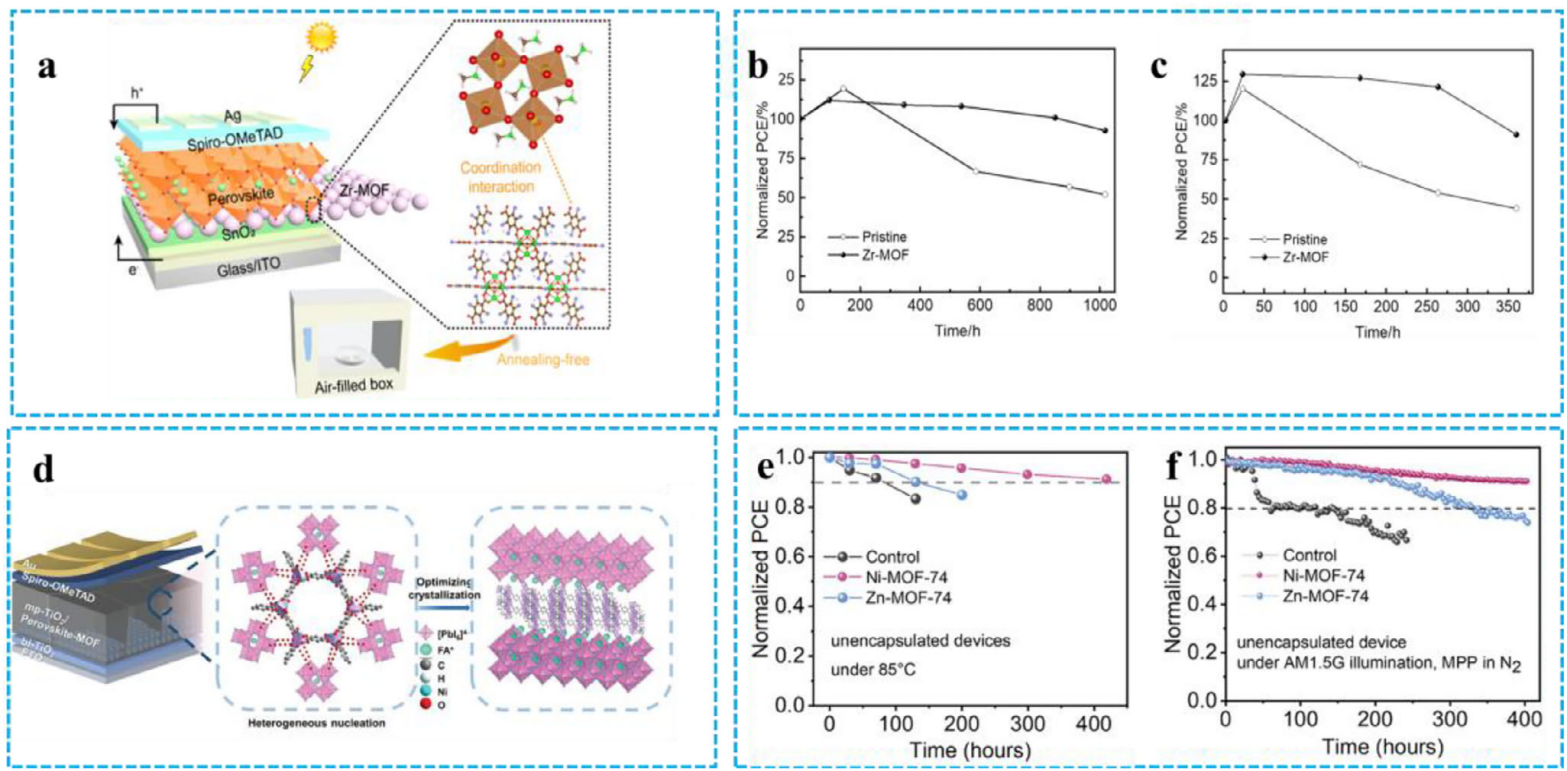
a) Diagrams depicting the PSCs composed of Zn–MOF/PVK, b) Assessment of air stability within an air‐filled enclosure and c) evaluation of thermal stability at 65 °C in a nitrogen atmosphere for the unsealed PSCs Reproduced with permission.^[^ ^85^ ^]^ Copyright 2023 American Chemical Society. d) Schematics of PSCs of Zn‐MOF/Ni‐MOF/PVK, e) Stability of heat of unencapsulated devices kept at 85 °C. f) Continuous Maximum Power Point (MPP) test. Reproduced with permission.^[^ ^27^ ^]^ Copyright 2024 Wiley.

An approach to precipitation without heating (annealing) for perovskite films has been devised by Luo et al. by leveraging the interface between tin oxide and perovskite via a Zr–MOF. The results demonstrate that even in the absence of heating, the Zr–MOF interaction can accelerate the synthesis of PeNCs and boost the conversion of perovskite precursors into PeNCs.^[^ ^85^ ^]^ Consequently, the trap density is reduced by approximately an order of magnitude, this is associated with substantial rises in the mobility of both electrons and holes, resulting in more efficient carrier extraction and reduced charge recombination. **Figure** **14**a illustrates the structure of the PSC, which is FTO/SnO_2_/perovskite‐Zn MOF/Spiro‐OMeTAD/Ag. Consequently, the J_EQE_ rises from 21.32 to 22.55 mA cm^−2^ at the Zr–MOF contact. This has resulted in the exchange of power efficiencies (PCE) of 20.24% for PSCs based on Zr–MOF, which is 2.2 times higher than that of the original PSCs (9.26%). Additionally, the Zr–MOF interfacial layer can greatly enhance PSCs' thermal and air stability. After storage in air for 1018 h, PSCs based on Zr–MOF showed 93% of their initial PCE, compared to 52% for the original PSCs (Figure 14b). After continuous heating at 65 °C for 360 h, PSCs based on Zr–MOF retained 91% of their initial PCE, while the original PSCs retained only 44% (Figure 14c). Chun et al. introduced two MOFs, amorphous Ni–MOF‐74 (amNi–MOF–74) (target 1) and crystalline Zn–MOF‐74 (crZn–MOF–74) (target 2), to regulate the crystallization of lead iodide.^[^ ^27^ ^]^ Compared to crZn–MOF–74, the incorporation of amNi–MOF–74 enabled rapid nucleation, leading to the formation of high‐quality perovskite films with large grain size and low trap density, which enhanced the charge transfer between the perovskite and the charge transport layers. The constructed PSC system structure, as shown in Figure 14d, is FTO/blTiO_2_/mp‐TiO_2_/FAPbI_3_/PEAI/Spiro‐OMeTAD/Au. PSGs modified with amorphous Ni–MOF–74 achieved a photovoltaic performance of 24.17%, exhibiting excellent thermal and humidity stability, and represent one of the highest‐performing PSCs integrated with MOFs. Integrated current densities of the control device, target device 1, and target device 2 calculated from the EQE are 24.1, 25.1, and 23.9 mA cm^−2^, respectively, indicating that target 1 has a better spectral response. Figure 14e shows the stability comparison of the control perovskite film, target 1, and target 2 at 25% RH storage condition. The thermal stabilities of these three devices were also measured by annealing devices in the vacuum oven at 85 °C. The target 1 device remained 91% of initial PCE after over 420 h, while control and target 2 devices dropped rapidly to 83% after 130 h and 85% after 200 h, respectively. For long‐term stability under continuous light soaking, all devices are unencapsulated and tested in the N_2_ glove box (Figure 14f). The amNiMOF–74 modified devices exhibit better light stability (90.9% of initial PCE) after over 400 h under ongoing AM1.5 illuminations by the highest power point tracking at 25 °C. However, the control devices and crZn–MOF–74 modified devices dropped down to 80% of the initial PCE after 70 and 300 h, respectively. The long‐time storage stability was carried out by unencapsulated devices under 25% RH in the dark. Although the porous structure and tunable chemical properties of MOFs can optimize the photovoltaic performance of PeNCs, such as improving light absorption efficiency and carrier mobility, thereby enhancing the PCE of solar cells, the PCE of PeMOFs solar cells is still lower than that of some traditional solar cell materials, such as single‐crystal silicon and PSCs. Further research and optimization are needed to improve their efficiency.

### 5.3 Photocatalysis

Upon light excitation, semiconductor materials generate electron‐hole pairs. These pairs diffuse to the material's surface, creating a strong redox potential that drives redox reactions. PeNCs, in particular, exhibit unique physical properties, including high extinction coefficients, narrow bandgaps, low exciton binding energies, and rapid charge transport, making them promising candidates for efficient photocatalysis.^[^ ^99^, ^100^, ^101^ ^]^ Single‐component PeNCs, however, suffer from rapid charge recombination of photogenerated electron‐hole pairs and relatively unstable chemical properties. The absence of effective catalytic sites further exacerbates these limitations, significantly hindering their photocatalytic efficiency. In contrast, PeMOFs, which integrate the benefits of both PeNCs and MOFs, offer a promising avenue for enhanced catalytic performance. This improvement can be achieved through strategies such as the design of specific metal sites, adjustment of doping bands, and optimization of ligands, effectively addressing the limitations of single‐component materials. Furthermore, the inherent advantages of MOFs and PeNCs, including efficient charge separation and transport, contribute to improved charge transfer efficiency, ultimately leading to enhanced photocatalytic efficiency.

**Figure 15:**
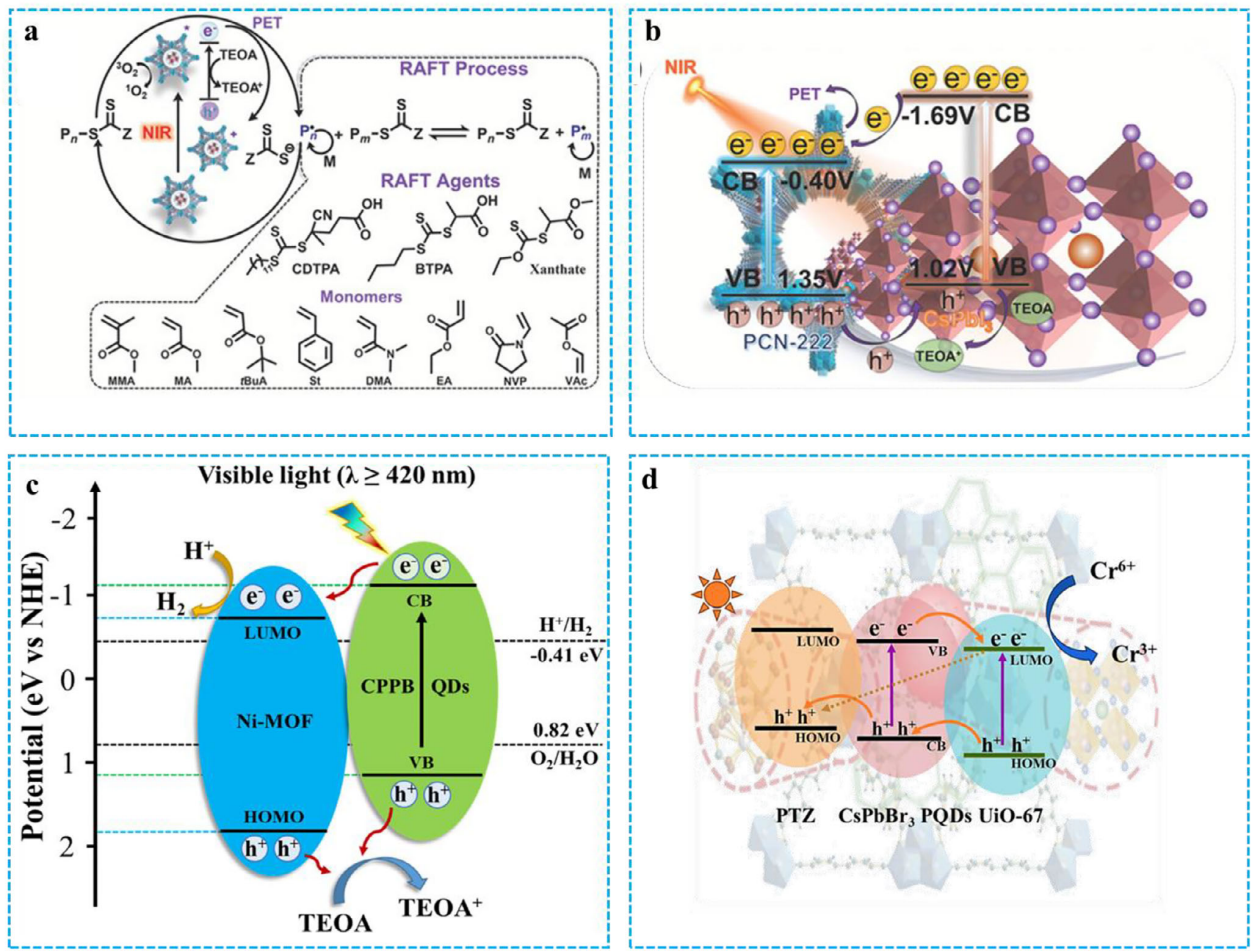
a) The PET–RAFT polymerization process facilitated by CsPbI_3_@PCN‐222 under NIR irradiation,^[^ ^25^ ^]^ b) an illustration of the energy gap structure and the processes of separation of charges and transfer across the CsPbI_3_@PCN‐222 photocatalyst caused by NIR light. Reproduced with permission.^[^ ^25^ ^]^ Copyright 2022 Wiley. c) A proposed mechanism for hydrogen evolution using CPPB@Ni‐MOFs as photocatalytic materials. Reproduced with permission.^[^ ^102^ ^]^ Copyright 2022 Elsevier. d) Energy alignment in CsPbBr_3_@UiO‐67@PTZ and the proposed photoinduced carrier transfer process at the interfaces Reproduced with permission.^[^ ^103^ ^]^ Copyright 2024 American Chemical Society.

Xia et al. introduced a combined approach, encapsulating PeNCs within a porphyrin‐like, zirconium‐based MOF (PCN‐222). Through systematic optimization, varying both the halide composition and the CsPbI_3_ content within the PCN‐222 matrix, they identified CsPbI_3_@PCN‐222 as the optimal photocatalyst. This material exhibits strong red and NIR light utilization and demonstrates resistance to oxygen and water, enabling reversible addition‐fragmentation chain transfer (RAFT) polymerization under photoinduced electron transfer (PET) conditions in both PET and polar organic solvents (**Figure** **15**a).^[^ ^25^ ^]^ PCN‐222 acts as a protective matrix, effectively shielding and stabilizing the in‐situ formed, environmentally sensitive CsPbI_3_ within its MOF channels. Furthermore, the extended channels of PCN‐222 promote the growth of CsPbI_3_ into a nanowire morphology. The resulting hybrid material, comprising perovskite nanowires integrated within the MOF structure, significantly reduces the diffusion length for photogenerated charge carriers, enhances charge separation efficiency, and improves red to NIR light absorption. As shown in Figure 15b, the valence bands (VB) of CsPbI_3_ and PCN‐222 are 1.02 and 1.35 V, respectively. Thus, it can be inferred that the electrons excited from perovskite are transferred to PCN‐222, while the photogenerated holes of PCN‐222 are transferred to perovskite. This binary mixed photocatalyst facilitated efficient PET‐RAFT polymerization of a diverse range of monomers, including both conjugated (e.g., styrene) and less reactive non‐conjugated monomers (e.g., vinyl acetate), under visible or NIR light irradiation. The catalyst demonstrated robust stability across multiple catalytic cycles, yielding high monomer conversion rates, narrow molecular weight distributions, and precise chain‐end control in the synthesized polymers. Moreover, the excellent performance of this binary photocatalyst allows it to leverage the deep penetration of NIR light, achieving high monomer conversion rates in photopolymerization even through significant barriers.

Zhang et al. synthesized Pt‐doped CsPbBr_3_ (CPPB) using the hot‐injection method. Subsequently, they fabricated 0D/2D CPPB@Ni‐MOF heterojunction photocatalysts by combining CPPB with two‐dimensional Ni–MOFs through van der Waals adsorption. The incorporation of Pt^2+^ not only enhanced the photothermal stability of CsPbBr_3_ but also broadened the possibilities for high‐activity photocatalysis, mitigating the environmental risks associated with Pb^2+^. Under illumination, CPPB acts as an electron donor, continuously supplying charges to the Ni–MOFs. This facilitates photocatalytic H_2_ generation, leveraging the large surface area and efficient charge carrier separation capabilities of the 2D Ni–MOFs. The proposed mechanism for hydrogen production is illustrated in Figure 15c.^[^ ^102^ ^]^ Initially, photoexcitation promotes electrons to the conduction band of CPPB. These electrons then migrate to the Ni–MOF surface through the charge transfer interface between the CPPB NCs and the Ni–MOFs. The accumulated electrons subsequently participate in the stable photocatalytic generation of H_2_. Throughout this process, the 2D Ni–MOFs provide abundant binding sites for CPPB, benefiting from their large surface area and unique structure. Furthermore, the incorporation of CPPB reduces the effective bandgap of the photocatalyst, broadening the light absorption range and accelerating charge carrier separation. The presence of Pt^2+^ also improves the catalytic activity and photothermal stability of CPPB, synergistically enhancing the overall photocatalytic performance of the catalyst. Similarly, Yin et al. utilized CsPbBe_3_ as electron donors and hole transfer mediators, while Zr‐based MOFs (UiO‐67) and pentazole (PTZ) acted as electron and hole acceptors, respectively. They constructed a photocatalyst with a dual heterojunction structure, based on the reported band structures, as depicted in Figure 15d.^[^ ^103^ ^]^ Compared to pure UiO‐67, the composite exhibited significantly improved photocatalytic activity for the reduction of Cr^6+^. The findings of this study may offer valuable insights for the design of photocatalysts for Cr^6+^ reduction and related applications.

In summary, MOFs, characterized by their porous structure and high specific surface area, offer abundant active sites that enhance photocatalytic performance. Moreover, the construction of MOFs heterostructures further boosts photocatalytic efficiency by promoting the separation of photoexcited charge carriers. Importantly, MOFs can also stabilize perovskite materials, shielding them from degradation caused by environmental factors such as moisture and oxygen during photocatalysis. This protective effect improves the catalyst's stability and extends its operational lifespan. Therefore, MOFs provide multifaceted advantages in perovskite‐based photocatalysis, leading to significant improvements in efficiency, stability, and selectivity, thus demonstrating substantial potential for widespread application.

### 5.4 Sensors

High‐sensitivity sensors are crucial in diverse fields such as environmental pollutant monitoring, medical diagnostics and control, and food safety analysis. These sensors rely on specialized materials that act as sensitive elements, detecting target analytes and converting their presence into measurable physical signals. Consequently, the performance of these sensor materials significantly impacts the overall sensitivity of the sensor.^[^ ^104^, ^105^ ^]^ PeMOFs hold significant potential for sensor applications, building upon the inherent sensing capabilities of both PeNCs and traditional MOFs. The detection of inorganic ion concentrations in water bodies is crucial for real‐time water quality assessment and the prevention of environmental pollution stemming from excessive ion levels. PeMOFs can be employed for ion detection due to their selective fluorescence quenching effect in the presence of specific metal ions. This mechanism relies on the interaction between metal ions and the luminescent centers within the PeMOFs structure, resulting in alterations in fluorescence intensity. These changes are directly correlated with the type and concentration of metal ions present in the surrounding environment, exhibiting a clear concentration‐dependent response.

**Figure 16:**
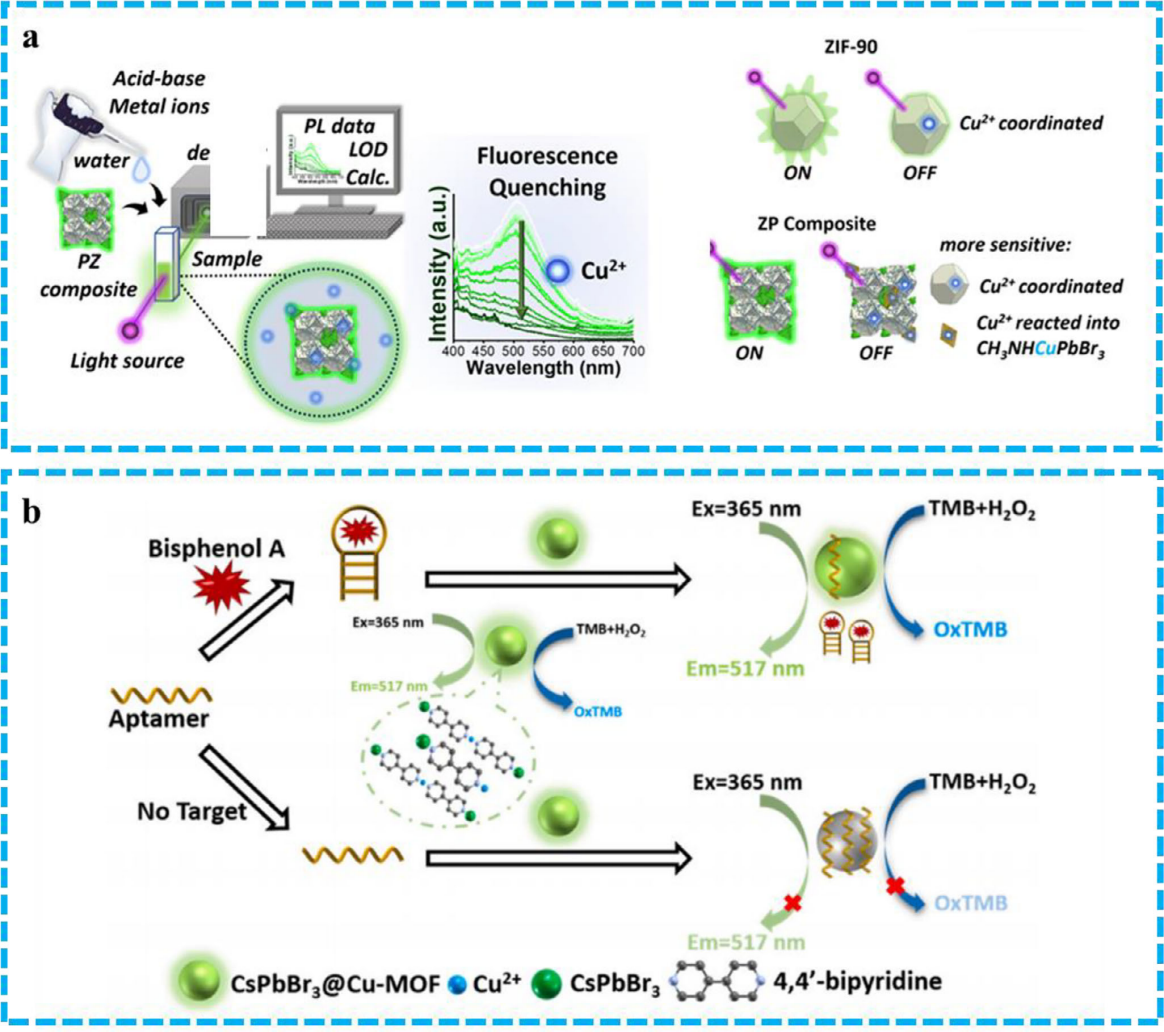
a) A diagrammatic representation of the mechanism by which the materials' fluorescence is enhanced by CH_3_NH_3_PbBr_3_ and subsequently quenched in the presence of Cu^2+^ ions. Reproduced with permission.^[^ ^87^ ^]^ Copyright 2024 American Chemical Society. b) A visual depiction of the dual‐functional sensing system that utilizes both fluorescent and colorimetric signals. Reproduced with permission.^[^ ^89^ ^]^ Copyright 2024 Elsevier.

Lai et al. introduced defects into the fluorescent ZIF‐90 framework by modifying the ligand‐to‐metal molar ratio.^[^ ^88^ ^]^ This defect engineering strategy aimed to generate additional void space to facilitate the incorporation of a greater quantity of PeNCs. The resulting composite material selectively detects Cu^2+^ ions in aqueous solution via a fluorescence quenching mechanism, as illustrated in **Figure** **16**a. Notably, both ZIF‐90 and CH_3_NH_3_PbBr_3_ exhibit green emission. The observed fluorescence enhancement in ZIF‐90 upon combination with CH_3_NH_3_PbBr_3_ can be attributed to two potential mechanisms. First, the green emission may originate predominantly from CH_3_NH_3_PbBr_3_ itself. Second, energy transfer from CH_3_NH_3_PbBr_3_ to ZIF‐90 could occur. The synthesized CH_3_NH_3_PbBr_3_@ZIF‐90 composites, in which CH_3_NH_3_PbBr_3_ is primarily located within the ZIF‐90 crystal layers and thus shielded from the aqueous solution, still allow Cu^2+^ ions to diffuse through the ZIF‐90 pores. These Cu^2+^ ions can then chelate with ZIF‐90 and/or interact with CH_3_NH_3_PbBr_3_, potentially leading to the formation of oxidized products. These oxidation processes result in a decrease in luminescence and subsequent fluorescence quenching. Furthermore, this system provides a platform to investigate ion diffusion kinetics, specifically relating to the ZIF‐90 pore dimensions and the kinetic radius of Cu^2+^ ions.

Beyond its use in ion sensing, PeMOFs has also found application in enzyme detection. For example, Ma et al. synthesized a CsPbBr_3_@Cu‐MOF exhibiting both fluorescent and chromogenic properties, employing 4,4′‐bipyridine (4,4‐b‐bpy) as an organic linker. The synthesis, achieved through an in‐situ formation and ligand‐assisted reprecipitation technique, allowed for facile adjustment of the ligand‐to‐metal mole ratio.^[^ ^89^ ^]^ 4,4′‐b‐bpy, acting as a bidentate ligand, coordinates with Cu^2+^ ions to form a Cu–MOF directly on the surface of CsPbBr_3_. This CsPbBr_3_@Cu‐MOF composite serves as a dual‐functional matrix, enabling both aptamer immobilization and signal transduction, resulting in a versatile sensing platform that utilizes both fluorescence and colorimetric detection. This approach simplifies and improves the sensitivity of bisphenol A (BPA) detection. Specifically, BPA reduces the aptamer's affinity for the CsPbBr_3_@Cu‐MOF by facilitating the formation of BPA‐aptamer complexes. Consequently, the CsPbBr_3_@Cu‐MOF exhibits restored fluorescence and gains peroxidase‐like activity (Figure 16b).

### 5.5 Anti‐Counterfeiting and Encryption

PeNCs, a novel class of highly luminous materials, have garnered significant attention in anti‐counterfeiting applications due to their tunable emission spectra, high luminescence intensity, and excellent photoluminescence stability. Furthermore, their unique optical properties, such as stimulus‐responsive fluorescence emissions triggered by light, heat, solvents, and mechanical forces, enable diverse anti‐counterfeiting strategies and enhanced information security. Notably, the encapsulation capabilities and inherent tunability of MOFs render PeNCs composites particularly promising for advanced anti‐counterfeiting and encryption technologies, surpassing the capabilities of PeNCs alone.

**Figure 17:**
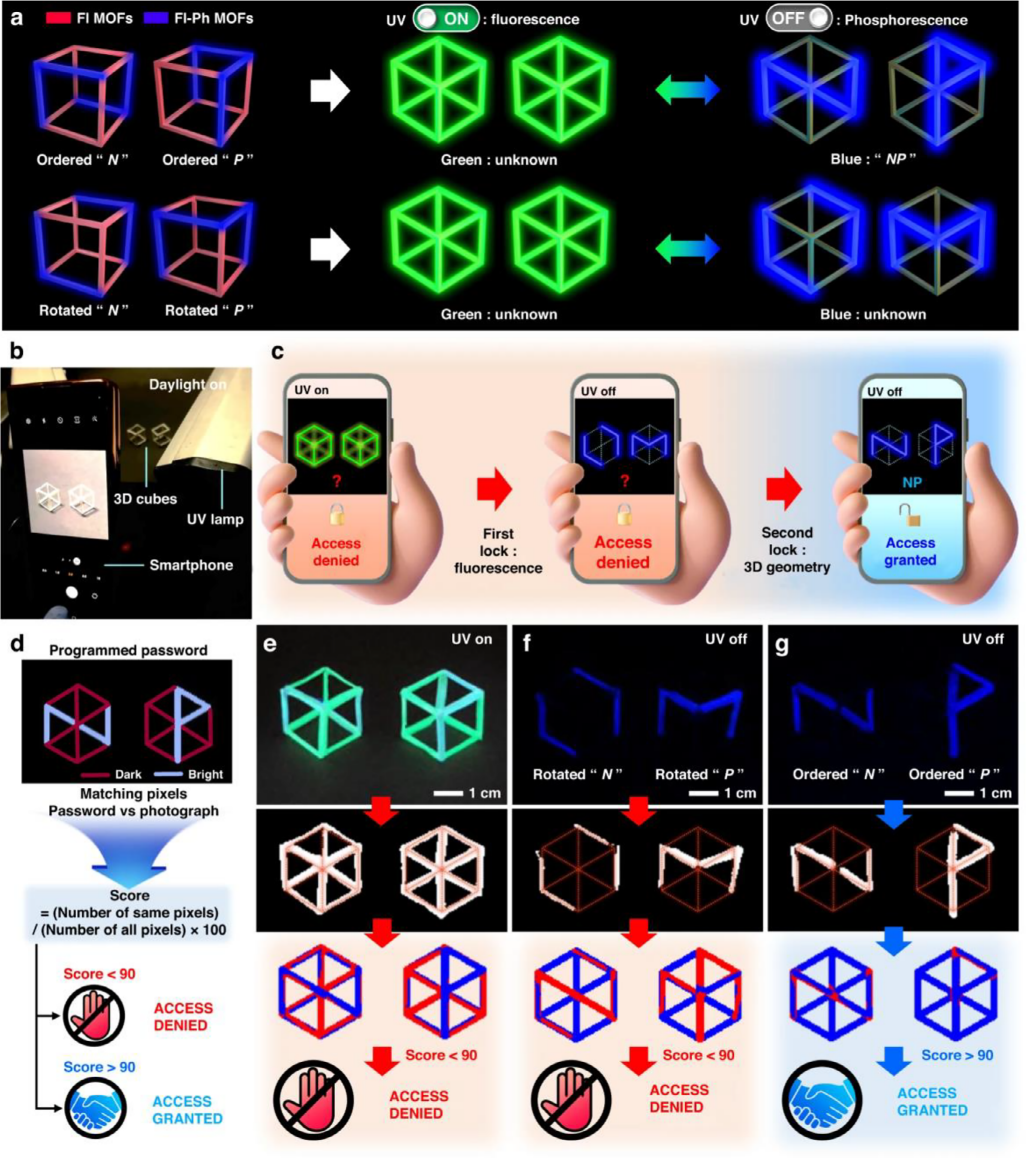
a) Enhancing the security of a cubic structure through a two‐tier 3D encryption process utilizing Fl‐PhMOF@PCL filaments. b) The procedure for operating a smartphone app to achieve this 3D double encryption with the aid of a standard camera. c) The method for decoding the encrypted data, which is concealed by blue phosphorescence beneath the fluorescence, via a smartphone application. d) The steps for deciphering the 3D encrypted content include e) employing bioluminescent cubes under UV illumination at 254 nm, f) manipulating the cubes once the UV source is extinguished, and g) aligning the cubes correctly post‐UV for the secure encoding of “NP”. Reproduced with permission.^[^ ^26^ ^]^ Copyright 2023 Springer Nature.

In this context, Oh et al. successfully integrated MOFs with PeNCs to create a novel encryption application.^[^ ^26^ ^]^ Specifically, they developed a 3D encryption technique using dual‐luminescent, printable phosphorescent metal‐organic frameworks (Fl‐PhMOFs). Their approach involved incorporating the PeNCs precursor methylammonium bromide (MABr) into lead‐containing MOFs that also included cyanuric acid (CA). This process facilitated the in‐situ synthesis of MAPbBr_3_ within the MOF structure. The resulting MAPbBr_3_ emits a characteristic green light under UV irradiation, endowing the composite material with dual‐photoluminescent properties, thus creating the Fl‐PhMOFs. The successful implementation of encryption and decryption for Fl‐PhMOFs‐based cubic structures via smartphone applications has opened avenues for developing advanced security systems for information encoding. As shown in **Figure** **17**a, two cubes were engineered, each containing a distinct blend of Fl‐PhMOFs embedded in polycaprolactone (PCL) and Fl‐MOFs embedded in PCL. These cubes were designed to display the ciphers “N” and “P” when illuminated from specific vertex angles. Under room‐temperature phosphorescence (RT‐OP) conditions, the combined cipher “NP” becomes visible from a particular viewpoint. A password validation method was developed, involving capturing RT‐OP emissions from both cubes using a standard smartphone camera (Figure 17b). Subsequent image analysis, performed using a custom‐built application, enabled the cubes to be used to unlock a conventional smartphone (Figure 17c). Upon exposure to UV radiation, the cubes exhibit strong fluorescence, revealing their hexagonal patterns. After identifying these patterns, the UV light is switched off to reveal the RT‐OP signatures. The smartphone camera then captures these RT‐OP images, which are analyzed and compared against the “NP” password for authentication (Figure 17d). Figure 17e–g demonstrate that access is granted only when the correct “NP” pattern, with its associated RT‐OP characteristics, is presented. This confirms that 3D encryption techniques, utilizing simple cubic structures made from dual‐luminescent MOFs/polymer composites, can be effectively integrated into a high‐security smartphone locking and unlocking system.

**Figure 18:**
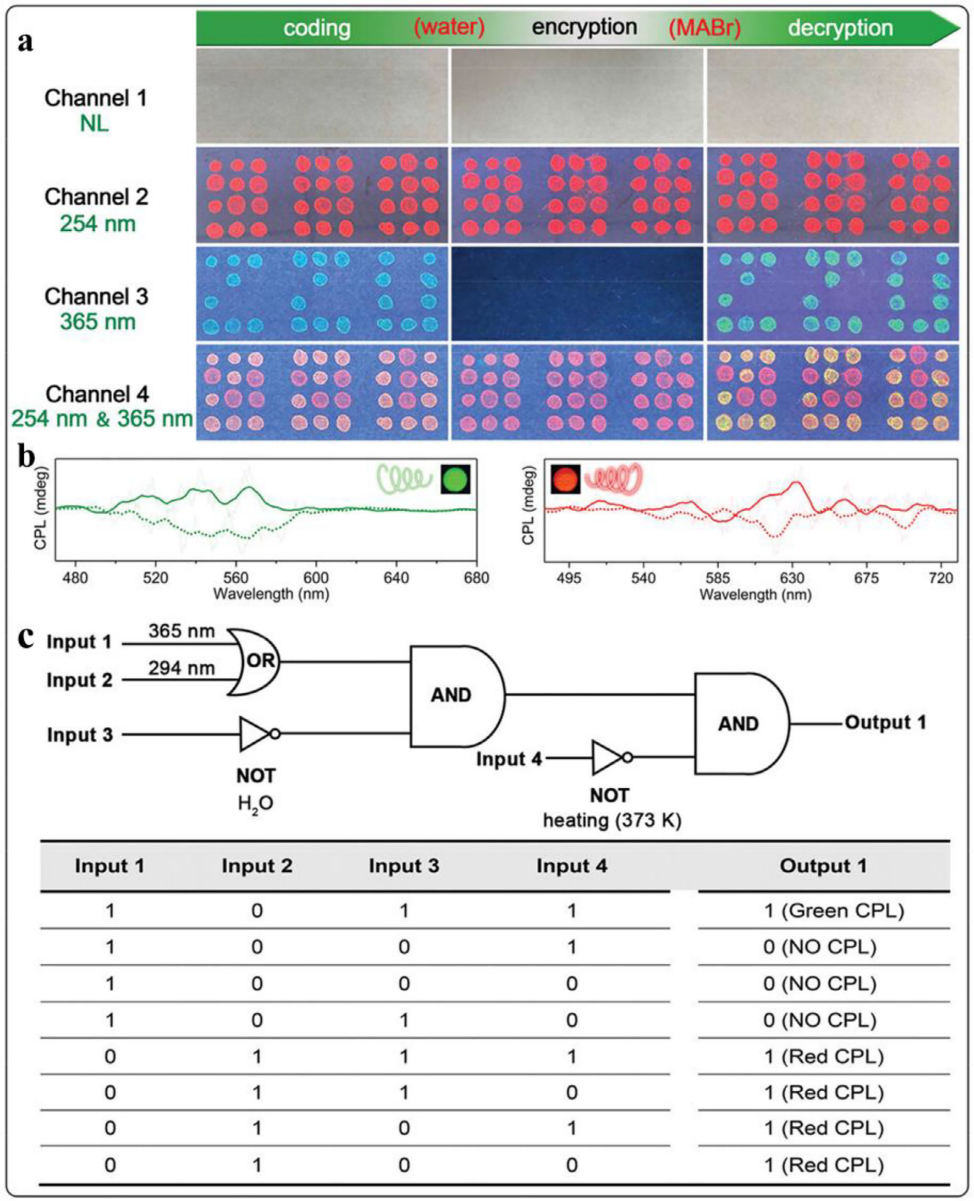
a) The process involves data encoding, encryption, and decryption using different light sources. b) The corresponding solid‐state CPL curves are shown for (P)–(+)–EuMOF@MAPbBr_3_ (solid lines) and (M)–(−)–EuMOF@MAPbBr_3_ (dashed lines). c) The integrated chiral logic gate's physical electrical representation, with inputs 1, 2, 3, and 4 represented by 365 and 294 nm wavelengths, H_2_O, and heating at 373 K, respectively. Output 1 corresponds to the CPL signal result. Reproduced with permission.^[^ ^64^ ^]^ Copyright 2021 Wiley.

Beyond phosphorescence, the PL and CPL properties of PeMOFs hold promise for anti‐counterfeiting and encryption applications. Zhang et al. demonstrated this potential in **Figure** **18**, showcasing reversible PL and CPL switching in (P)–(+)/(M)–(−)–EuMOF@MAPbBr_3_ composites for information encoding, encryption, and decryption.^[^ ^64^ ^]^ These composites were used to create a “ZZU” pattern on filter paper, demonstrating information encoding, encryption, and decryption through various stimuli‐responsive behaviors. As shown in Figure 18a, the “ZZU” pattern was encoded by selectively depositing an ethanol solution of (M)–(−)–EuMOF@MAPbBr_3_ (orange) and (M)–(−)–EuMOF@PbBr_2_ (white) composites onto designated circular areas of the filter paper. After drying, the (M)–(−)–EuMOF@MAPbBr3 circles appeared pale yellow, forming the “ZZU” symbol. The (M)–(−)–EuMOF@PbBr_2_ circles, however, were not visible under ambient light. Upon exposure to a 254 nm UV lamp (Channel 2), all circles emitted red light, effectively concealing the “ZZU” pattern. Conversely, when a 365 nm UV lamp was used (Channel 3), only the “ZZU” pattern emitted green light. Simultaneous activation of both the 254 and 365 nm UV lamps (Channel 4) resulted in a color change of the “ZZU” pattern's emission from greenish to yellow, while the remaining circles fluoresced pink. Figure 18b illustrates the circularly polarized luminescence (CPL) characteristics of the (P)–(+)/(M)–(−)–EuMOF@MAPbBr_3_ composites. Two distinct CPL spectra were observed, corresponding to (P)–(+)–EuMOF@MAPbBr_3_ and (M)–(−)‐EuMOF@MAPbBr_3_, respectively. These composites exhibit opposite CPL signals, indicating complementary chirality, which can be exploited for developing sophisticated encryption schemes. Figure 18c presents a novel concept of a chiral‐integrated combinatorial logic gate based on the (P)–(+)/(M)–(−)–EuMOF@MAPbBr_3_ composites. External stimuli (365, 294 nm, H_2_O, and heating at 373 K) serve as inputs, and the CPL signals act as outputs. Input 1 (365 nm) activates the green CPL of (P)–(+)/(M)–(−)–EuMOF@MAPbBr_3_. Output 1 produces the corresponding CPL signal (logic value, 1) only when Inputs 3 and 4 (H_2_O and heating at 373 K) are both inactive (logic values, 1 and 1); otherwise, it is CPL‐silent (logic value, 0). When Input 2 (294 nm) is activated, Output 1 generates red CPL (logic value, 1), regardless of the state of Inputs 3 and 4. This demonstrates the effective implementation of an implication logic gate. In summary, the PeMOFs can achieve distinct and reversible switching of signals under fluorescence and phosphorescence. This suggests significant potential for anti‐counterfeiting applications.

### 5.6 Scintillators

PeNCs have become compelling scintillators for X‐ray detection and imaging due to their advantageous properties, including high quantum efficiency, rapid decay times, enhanced X‐ray absorption, affordability, and facile crystal growth.^[^ ^106^, ^107^ ^]^ MOFs can facilitate the in situ, spatially confined formation of PeNCs, simultaneously providing quantum confinement, surface passivation, and protection. This host‐guest approach leads to high exciton binding energy and bright, efficient luminescence, which significantly enhances the stability of PeNCs against degradation from air, heat, UV, solvents, and X‐ray irradiation. Consequently, the MOFs/PeNCs composite scintillators are a promising approach for developing advanced X‐ray detectors.

**Figure 19:**
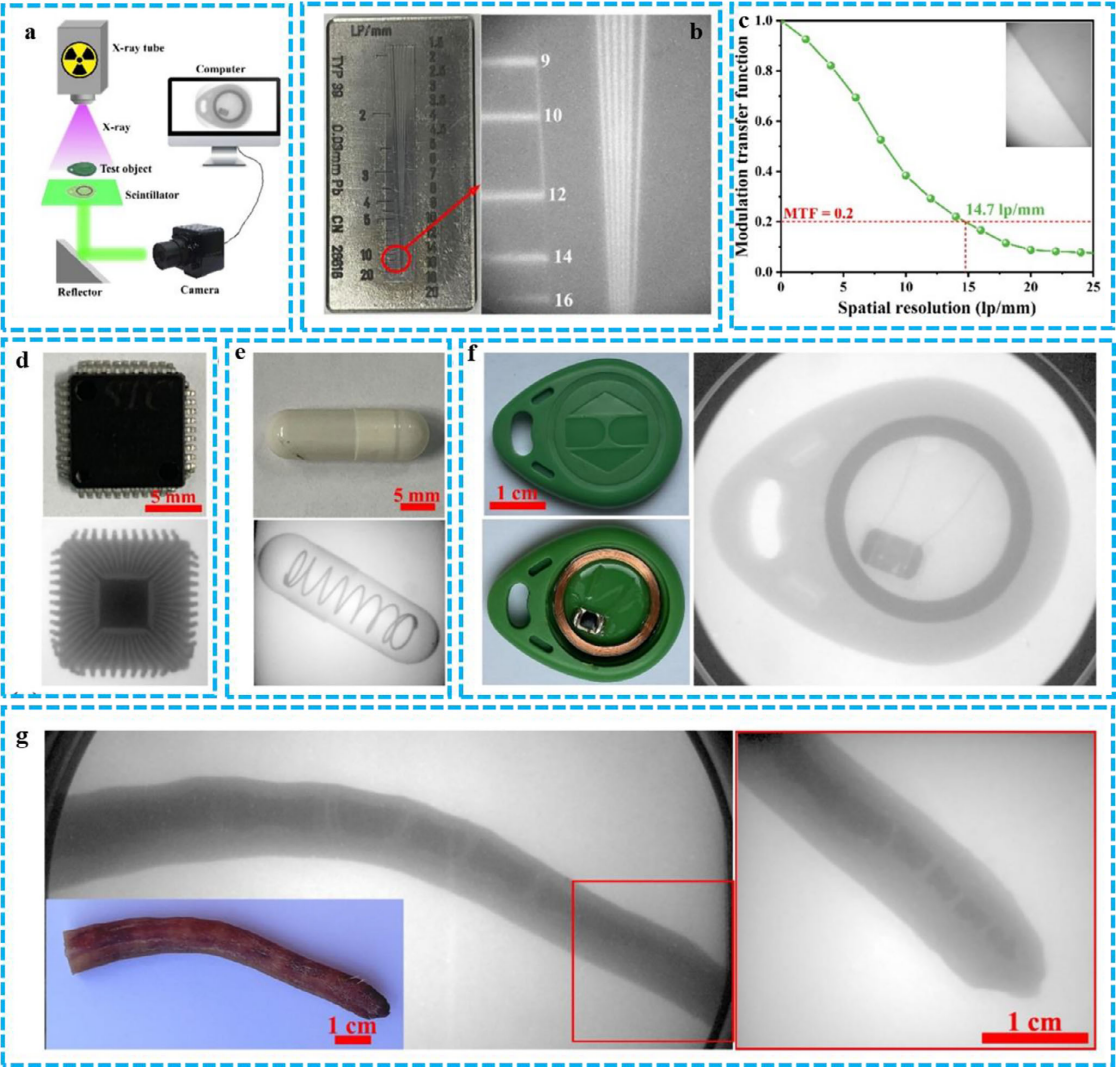
a) Diagram of the X‐ray imaging apparatus. b) Snapshot of the standard X‐ray pattern plate along with the X‐ray image of a section of the pattern plate. c) MTF curves derived from a metallic edge using bMOF@MAPbBr_3_‐PMMA composite films. Visual representations of d) a microchip, e) a capsule with an internal spring, f) an electronic lock, and g) a pigtail with its corresponding X‐ray image captured by bMOF@MAPbBr_3_‐PMMA composite films. Reproduced with permission.^[^ ^93^ ^]^ Copyright 2024 Elsevier.

Wu et al. employed a host‐guest spatial confinement technique to create a novel and reliable scintillator with high performance for X‐ray imaging. This technique involved encapsulating MAPbBr_3_ within the bio‐MOF‐100 host framework (bMOF)@MAPbBr_3_ using a two‐stage deposition method.^[^ ^93^ ^]^ Utilizing the advantages of surface passivation and quantum confinement, this material exhibits rapid PL decay, a high PLQY of almost 60%, and bright green light. Additionally, bMOF@MAPbBr_3_ shows much‐improved resistance to deterioration from polar solvents, heat, moisture, air, UV light, and X‐ray irradiation. It has a linear X‐ray response because of its high energy of exciton binding, excellent optical properties, and improved firmness. For X‐ray scintillation, the material's internal quantum efficiency is comparable to that of the market scintillator LuAG, and its ultra‐low X‐ray detection threshold is 0.17 µGyair^−1^. Moreover, the bMOF@MAPbBr_3_‐PMMA scintillating screen delivers high spatial resolution, reaching up to 14.7 lpmm^−1^ and MTF = 0.2. The X‐ray imaging setup is illustrated in **Figure** **19**a using the bMOF@MAPbBr_3_‐PMMA scintillator. The spatial resolution was assessed by capturing a standard X‐ray resolution test pattern plate image as shown in Figure 19b, with the resolution limit found to be around 14–16 lpmm^−1^. The modulation transfer function (MTF) curve was then calculated using the slanted edge method on an aluminum sheet with a sharp edge, as seen in Figure 19c. This figure shows that the bMOF@MAPbBr_3_‐PMMA scintillating screen has a spatial resolution of 14.7 lpmm^−1^ (MTF = 0.2), aligning with the observed resolution limit. Due to increased light scattering in thicker films, the spatial resolution (MTF = 0.2) steadily drops from 14.7 to 9.6 lpmm^−1^ as the screen thickness grows from 35 to 1000 µm. Furthermore, X‐ray pictures of a variety of everyday objects were obtained using the bMOF@MAPbBr_3_‐PMMA scintillator. Figure 19d demonstrates that placing a small chip on the scintillating screen results in a clear X‐ray image of the chip's internal structure, with precise metal support distribution, indicating the scintillator's high spatial resolution and contrast. A clear X‐ray image of a spring‐encased capsule is presented in Figure 19e. The interior metal structure and circuitry of an electronic key with a plastic shell are visible in the X‐ray image shown in Figure 19f. Similarly, Figure 19g shows that even with an enlarged screen, the internal small bones of a pig's tail are easily visible under X‐ray imaging, validating the scintillator's capacity for detailed imaging. At present, the research on PeMOFs for X‐ray applications is relatively limited, which may become a key research direction in the future.

## 6 Conclusion and Prospects

This review systematically summarizes the synthesis, performance, and applications of PeMOFs composites. It focuses on two primary preparation strategies: the “ship‐in‐a‐bottle” and “bottle‐around‐the‐ship” methods. The discussion highlights the performance enhancements achieved through these strategies, including stability improvement, performance optimization, tunable luminescent properties, and controlled growth using MOFs as templates. Building upon this foundation, the review summarizes the significant progress made by PeMOFs in diverse applications, including solar cells, LEDs, photocatalysis, fluorescent anti‐counterfeiting, sensors, and information encryption. Despite the rapid advancements in this field, several challenges and unresolved issues remain. These can be summarized as follows:

The mismatch between the pore structure of MOFs and the size and geometric shape of PeNCs limits the performance of composites, which remains one of the main obstacles to the development of PeMOFs. Based on the two existing synthetic strategies, there is an urgent need to develop more innovative synthetic methods to promote progress in this field. For example, it is necessary to explore how to avoid introducing too many polar solvents during the “bottle‐around‐the‐ship” synthesis procedure to increase the composites' endurance.

Most PeNCs materials contain toxic lead, which may lead to environmental pollution issues in practical applications. Although the preparation and application of lead‐free PeNCs composites have attracted more and more attention, this field still requires more research investment. For instance, CsSnBr_3_, as an emerging lead‐free PeNCs, its potential in the preparation of PeMOFs and applications in various fields is in urgent need of further exploration.

Currently, many research works focus on fields such as photovoltaic cells, photocatalysis, LEDs, information security, and sensors. However, in addition to radiation detection and scintillators mentioned in this review, many organo‐inorganic PeNCs have become promising materials for chiral electronics, spintronics, and ferroelectrics. Compared with low‐dimensional chiral PeNCs, theoretically superior 3D chiral PeNCs have made slow progress due to stability issues. Combining 3D chiral PeNCs with MOFs is expected to solve this bottleneck and promote the development of chiral optoelectronic devices.

In recent years, the functionality of PeMOFs has been explored to some extent, but in practical applications, the unique advantages of MOFs have not been fully utilized. At the same time, there is a lack of research on surface modification of MOFs after the introduction of metal ions to improve the performance of PeMOFs. The mechanisms of unique properties of composites in many fields have not been fully elucidated, for example, the role of PeMOFs in the hydrogen evolution reaction process has not been explained, which greatly limits their further research. Therefore, more potential roles and verification work of MOFs need to be carried out. It is hoped that more research teams will investigate the crystal structure, synthetic methods, and performance of composite materials to better promote the development of PeMOFs. How to rationally select MOFs and PeNCss to achieve synergistic enhancement of PeMOFs performance has become an important direction for the development of this field. In previous years, machine learning technology has gradually become an important tool for material screening. It is anticipated that using machine learning to forecast the composition and functionality of PeMOFs may hasten the identification of high‐performance PeMOFs and provide scientific guidance for their rational design, thus promoting further development in this field.

In the next research, advanced tech like Electron Energy Loss Spectroscopy (EELS) can be used for micro‐area analysis. This helps deeply study the interface structure, atomic arrangement, and element distribution between MOFs and perovskites, revealing the link between the composite material's microstructure and performance. Also, in‐situ characterization tech should be developed and applied. For example, in‐situ X‐ray diffraction (in‐situ XRD), in‐situ Raman spectroscopy, and in‐situ X‐ray photoelectron spectroscopy (in‐situ XPS) can monitor the material preparation process in real‐time. These techs offer deep understanding of material formation and dynamic evolution and guide synthesis strategy optimization.

In addition to this, in the screening process, AI models can learn from existing MOF and perovskite data to build efficient screening models. These models can rapidly identify promising MOF/perovskite combinations from vast material libraries, improving screening efficiency while discovering unique combinations that traditional manual methods might miss. This expands the scope of materials for research and application and increases the chances of finding high‐performance hybrid systems.

For optimization, machine learning can use experimental data and simulation results to intelligently optimize the preparation parameters and composition ratios of MOF/perovskite hybrid systems. By analyzing large amounts of data and building correlation models, it can pinpoint the key factors affecting performance and suggest optimal parameter combinations. This achieves precise performance control of the hybrid systems, improves their indicators to better meet application requirements, and drives continuous advancement of MOF/perovskite hybrid systems in both performance and application.

In summary, PeMOFs have broad prospects but also many challenges to be solved. However, with further development and exploration of interdisciplinary fields, PeMOFs with good stability, unique functions, and a wide range of applications will gradually appear in various important application fields. It is hoped that more research teams will investigate the connection between the makeup of crystalline, synthetic methods, and the performance of composite materials to better promote the development of PeMOFs.

## Conflict of Interest

The authors declare no conflict of interest.
